# MAESTRO - multi agent stability prediction upon point mutations

**DOI:** 10.1186/s12859-015-0548-6

**Published:** 2015-04-16

**Authors:** Josef Laimer, Heidi Hofer, Marko Fritz, Stefan Wegenkittl, Peter Lackner

**Affiliations:** 1 Department of Molecular Biology, University of Salzburg, Hellbrunnerstr, Salzburg, 34, 5020 Austria; 2 University of Applied Sciences Upper Austria, School of Informatics, Communications and Media, Softwarepark 11, Hagenberg, 4232 Austria; 3 Salzburg University of Applied Sciences, Urstein Süd 1, Puch, 5412 Austria

**Keywords:** Protein stability, Point mutation, Stability prediction, Machine learning, Statistical energy function

## Abstract

**Background:**

Point mutations can have a strong impact on protein stability. A change in stability may subsequently lead to dysfunction and finally cause diseases. Moreover, protein engineering approaches aim to deliberately modify protein properties, where stability is a major constraint. In order to support basic research and protein design tasks, several computational tools for predicting the change in stability upon mutations have been developed. Comparative studies have shown the usefulness but also limitations of such programs.

**Results:**

We aim to contribute a novel method for predicting changes in stability upon point mutation in proteins called MAESTRO. MAESTRO is structure based and distinguishes itself from similar approaches in the following points: (i) MAESTRO implements a multi-agent machine learning system. (ii) It also provides predicted free energy change (*Δ* *Δ*G) values and a corresponding prediction confidence estimation. (iii) It provides high throughput scanning for multi-point mutations where sites and types of mutation can be comprehensively controlled. (iv) Finally, the software provides a specific mode for the prediction of stabilizing disulfide bonds. The predictive power of MAESTRO for single point mutations and stabilizing disulfide bonds is comparable to similar methods.

**Conclusions:**

MAESTRO is a versatile tool in the field of stability change prediction upon point mutations. Executables for the Linux and Windows operating systems are freely available to non-commercial users from http://biwww.che.sbg.ac.at/MAESTRO.

**Electronic supplementary material:**

The online version of this article (doi:10.1186/s12859-015-0548-6) contains supplementary material, which is available to authorized users.

## Background

Point mutations can have a strong effect on the thermodynamic stability of proteins. In consequence, this change in stability may have an impact on the protein’s function and subsequently may cause diseases [1]. Deliberately increasing the stability of a protein or keeping it stable while changing certain other protein properties is often a goal in biotechnology, e.g. to optimize industrial processes, or also in drug design or basic research. Experimentally, directed molecular evolution [2] is a valuable tool for designing such stable variants. Also rational design may lead to the desired results. However, wet lab approaches are costly and time consuming endeavors.

The need of powerful *in-silico* methods to predict the effect of point mutation on the protein stability is obvious. Therefore, several tools have been developed in the last two decades. They can roughly be divided into sequence based and structure based methods, where the latter implicitly also use sequence information. Sequence based methods such as MuStab [3] or iPTREE-STAB [4] usually utilize machine learning approaches such as support vector machines, neural networks or decision trees. Their advantage is that no structure is required. However, their performance is rather limited. I-mutant2.0 [5] and MUpro [6] operate sequence based in principle but can include structural information if available. Structure based tools AUTO-MUTE [7], CUPSAT [8], Dmutant [9], FoldX [10], Eris [11], PoPMuSiC [12], SDM [13] or mCSM [14] usually perform better than the sequence based counterparts. Recently, SDM and mCSM have been integrated into a new method called DUET [15], which further improves the predictive power compared to the single methods. Stability prediction methods can also be divided into classifiers, which predict if a mutation is stabilizing or destabilizing, or into *Δ* *Δ*G predictors which aim to quantify the extent of the (de)stabilization. The performance of different predictors regarding classification and *Δ* *Δ*G values was investigated by Khan and Vihinen [16] and Potapov *et al.* [17] respectively. The authors of both studies indicate that the methods are useful but also that there is still room for improvements.

Structure based methods require a 3d structure of the wild type protein in order to perform the prediction, which poses a major restriction in their applicability. However, Gonnelli *et al.* [18] applied the PoPMuSiC predictor to a series of models of different degree of accuracy. They have shown, that even models built on remote homologs can be used as input for PoPMuSiC without a substantial loss of predictive power. The wild type structure does not necessarily need to be experimentally resolved.

The goal of our study was to improve the accuracy, robustness and range of applicability of change in stability predictions in the structure based *Δ* *Δ*G delivering method category. For this purpose we (i) implemented statistical scoring functions (SSF, also known as statistical energy function, SEF) as the main prediction component, (ii) defined an ensemble prediction strategy employing various concepts from the field of machine learning, (iii) assembled a clean training data set from ProTherm database [19], (iv) tested and validated our method on the basis of this data and other widely used data sets, and (v) compiled an easy to use standalone program which provides different kind of mutation experiments on single chains and protein complexes.

Below we describe the SSFs and the particular parameters optimized for the application in stability prediction and we report the performance of a pure SSF based approach. To further improve the predictive power we combine multiple linear regression (MLR), a neural network approach (NN) and a support vector machine (SVM) to a multi-agent method. This allows to incorporate additional sequence and structure information such as protein size or solvent accessibility, which proofed to be useful by several authors [5,12]. Technically, our approach integrates the concept of ensemble predictors [20] and multi-agent systems. We name the different prediction components agents. Derived from the different agent predictions, our method delivers a confidence estimation for each change in stability prediction. The performance of MAESTRO is compared to PoPMuSiC and mCSM, our main competitor methods.

In terms of applicability MAESTRO facilitates the investigation of particular chains or biological assemblies as provided by PDB and the analysis of a whole NMR ensemble in a single run. An easy to use mutation selection syntax allows to specify mutation sites by amino acid types, positions or solvent accessibility and the replacement residue by amino acid type. We added a brute force method, a greedy method and an evolutionary algorithm based method to search for multiple point mutations. The MAESTRO software is available as executable for Linux and Windows.

## Methods

**Figure 1:**
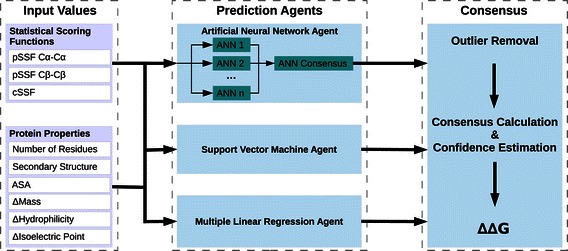
Scheme of MAESTRO’s main components and data flow.

MAESTRO is a multi-agent prediction system, based on statistical scoring functions (SSFs) and different machine learning approaches. First, we discuss the input values and the design of MAESTRO, followed by a description of the training strategies and data sets used for this work. A scheme of MAESTRO’s components and data flow is shown in Figure 1.

### Input values

For all agents nine input values have been used. They divide into two classes: (i) statistical scoring functions and (ii) protein properties such as size or environment at the mutation site.

#### Statistical scoring functions

We implemented two types of statistical scoring functions, also known as knowledge-based potentials or potentials of mean force. The first type are distance-dependent residue pair SSFs (pSSFs) as described by Sippl [21,22]. Here, *C* ^*α*^- *C* ^*α*^ and *C* ^*β*^- *C* ^*β*^ SSFs are considered. The second type of SSFs capture the solvent exposure of protein residues (cSSFs), by scoring the contacts of *C* ^*α*^ atoms within a defined radius around each residue represented by its *C* ^*α*^ atom [22].

To comply with the terminology of earlier publications [22] we call the contribution of a certain pair interaction an energy, defined as:

(1) $$  E_{ab}^{o}(d) = -ln\left(\frac{\delta_{ab}(d)}{\rho(d)}\right)  $$

where *δ*_*ab*_(*d*) is, e.g. in case of pSSFs, the relative frequency for the observed distance *d* for a certain pair (*a*,*b*) of amino acids, and *ρ*(*d*) is the relative frequency in the reference distribution.

The contact SSF is defined as:

(2) $$  {E_{a}^{o}}(c) = -ln\left(\frac{\delta_{a}(c)}{\rho(c)}\right)  $$

where *a* denotes a certain type of amino acid, *c* denotes the number of *C* ^*α*^ atoms within the contact radius, *δ*_*a*_(*c*) is the relative frequency of *c* for amino acid *a* and *ρ*(*c*) is the relative frequency in the reference distribution. The reference distribution *ρ* in equations (1) and (2) is the sum over all single distributions *δ*_*ab*_(*d*) and *δ*_*a*_(*c*) respectively.

For a certain protein, the $E_{\textit {ab}}^{o}(d)$ values for all interactions are summed up to the total Energy *E*. In order to use *E* to compare different sequences in one conformational state, which is the case when we introduce point mutations, *E* needs to be normalized with respect to a background model [21,23]. The same holds true for all different pSSFs and cSSFs.

First, an expected value $E_{\textit {ab}}^{e}$ for each observed energy $E_{\textit {ab}}^{o}$ has to be calculated. Here, $E_{\textit {ab}}^{e}$ is defined as the average energy that would be reached in the before mentioned set of known structures and for the given interaction type (*a*,*b*). Let *f*(*d*) be the frequency of all observed distances *d* between the representative atoms. Then, the expected value is:

(3) $$ E_{ab}^{e} = \frac{\sum_{d}{\left(E_{ab}(d) f(d) \right) }}{\sum_{d}{f(d)}}  $$

Similar, the expected value for the contact energy ${E_{a}^{e}}$ for a certain amino acid *a* is then:

(4) $$ {E_{a}^{e}} = \frac{\sum_{c}{\left(E_{a}(c) f(c) \right) }}{\sum_{c}{f(c)}}  $$

where *c* is the number of contacts and *f*(*c*) is the distribution of all observed contacts.

Among the statistics suitable for detecting shifts of the mean of distributions, the statistic given in equation (6) used in the Wilcoxon-Mann-Whitney-Test (U-Test, for short) is well-known for its power in case of heavy-tailed distributions [24]. Calculating p-values would rely on additional assumptions on the data and is not applicable here. Instead, we only use the statistic as a discriminative score for the deviation of means. Among several alternatives, the score function (6) performed best in our empirical tests. Therefore, we first rank the union of both sets of energies $E_{\textit {ab}}^{o}$ and $E_{\textit {ab}}^{e}$. Then, the rank sum *W* of the *n* observed energies as well as the expected rank mean *μ*_*W*_ and standard deviation *σ*_*W*_

(5) $$ \mu_{W} = \frac{n\left(2n+1\right)}{2} ~~~~~~~~ \sigma_{W} = \sqrt{\frac{n^{2}\left(2n+1\right)}{12}}   $$

are used to calculate a z-score like quantity:

(6) $$ s = \frac{W-\mu_{W}}{\sigma_{W}}   $$

The score is computed independently for the two pSSFs (*C* ^*α*^−*C* ^*α*^,*C* ^*β*^−*C* ^*β*^) and the cSSF. The three scores (*s*_*pSSFα*_,*s*_*pSSFβ*_,*s*_*cSSF*_) act as the first three input values for the prediction agents. In addition the combined score *s*_*c*_, based on the three scores, can be used as alternative stability change predictor. For this purpose we use Stouffer’s z-score method:

(7) $$ s_{c} = \frac{s_{\textit{pSSF}\alpha} + s_{\textit{pSSF}\beta} + s_{cSSF}}{\sqrt{3}}  $$

#### Protein properties

The SSFs are complemented by a selection of global and local protein properties. As global property we use the size of the protein. The local environment at the mutation site is described by the secondary structure state and the accessible surface area (ASA). The secondary structure assignment is performed with a refined version of the SABA [25] algorithm. The ASA computation resembles an adaption of the Geometry library algorithm [26]. The substitution is described by the change in mass, hydrophilicity and isoelectric point. In sum, protein size, secondary structure state and ASA together with *Δ*mass, *Δ*hydrophilicity and *Δ*isoelectric point are the remaining six input values for the prediction agents.

### Prediction agents

MAESTRO includes prediction agents on the basis of two different machine learning approaches, namely artificial neural networks (ANN) and support vector machines (SVM), and also on multiple linear regression (MLR).

The ANN agents use OpenNN [27], an open source C++ library. In order to improve generalization, a set of ANNs is utilized rather than a single one. An individual ANN consists of an input layer with nine nodes, one hidden layer and an output layer with a single node. The number of nodes in the hidden layer is optimized during training. Each ANN in this set is trained on different data. Details are explained below in section “Agent training”. In the prediction phase, their outputs are averaged. The SVM agents employ libSVM [28] and use an *ε*-SVR with a Gaussian kernel. The prediction parameters for the multiple linear regression agents are computed using the MLR implementation provided by the GNU Scientific Library [29].

In addition to their underlying prediction method, the agents differ on their specialization. We distinguish general agents with no specialization and agents specialized on substitutions which stabilize or destabilize a protein respectively. The latter ones are trained on either stabilizing or destabilizing mutations. During prediction, the general agents first predict if a mutation is stabilizing or destabilizing. Subsequently, the corresponding specialists are utilized. A flowchart of this process is provided in supplementary Figure S1 in Additional file 1. There, we also report data on the impact of the specialized agents. Overall, MAESTRO employs seven agents (three ANN agents, three SVM agents and one MLR agent). All seven agents are used in all experiments reported below in the same way for all proteins and all types of mutations.

### Prediction consensus and confidence estimation

MAESTRO performs a prediction in three main steps: (i) the computation of the SSF based score and the other input values, (ii) the prediction by the agents, and (iii) finally, the calculation of a consensus prediction and a corresponding confidence score.

Between the steps (ii) and (iii) outlier values of distinct agent predictions are removed iteratively. In each iteration the prediction value which differs most from the mean *μ*_*p*_ of the other predictions is removed, if the difference is larger than two standard deviations. This procedure is repeated as long as an outlier can be found or only two prediction values are left. The remaining agent values are then averaged for the final prediction value.

During the performance tests, we observed that the standard deviation of the agents predictions *σ*_*r*_ correlates with the prediction error. Therefore we implemented an *ad hoc* solution, based on *σ*_*r*_ to provide a confidence estimation (not to be confused with a confidence interval in the statistical sense) for MAESTRO’s *Δ* *Δ*G predictions. For an easy interpretation of this measure we relate *σ*_*r*_ to a cutoff *σ*_*max*_. The cutoff is defined as four times the standard deviation of the experimental determined *Δ* *Δ*G values in the training sets. A normalized confidence estimation *c*_*pred*_ is the calculated as:

(8) $$\begin{array}{@{}rcl@{}} c_{\mathit{pred}} = \left\{ \begin{array}{ll} 0 & \qquad\qquad\quad \text{if}\; \sigma_{r} > \sigma_{\mathit{max}}\\ 1 &- \left(\frac{\sigma_{r}}{\sigma_{\mathit{max}}}\right) \quad \text{if}\; \sigma_{r} \leq \sigma_{\mathit{max}} \end{array} \right.  \end{array} $$

The confidence estimation is numerically confined to values between 0.0 and 1.0, where 1.0 corresponds to a perfect consensus of all agents. As shown in the results, *c*_*pred*_ provides a sound estimation of the prediction accuracy.

### Agent training

All agents are trained independently from each other, but on the same input data set. As the agent relying on linear regressions does not depend on a special strategy, only the training strategies for the ANN and SVM agents are explained bellow.

#### ANN agents

An ANN agent utilizes a set of ANNs to perform its predictions. Each of these ANNs is trained on a subset of the input data set. For this, the training set is partitioned into ten subsets. Following a 10-fold cross validation, one of these subsets is used as generalization set, one is used as test set, and the remaining eight subsets are used for training. The best performing ANNs obtained during cross validation are finally utilized for the predictions. This reduces the risk of overfitting while no training data are being wasted.

When comparing the performance of MAESTRO to the competitor methods, the k-fold cross validation is adapted as follows: The test set is fixed in a certain fold for the whole set of ANNs and the training is performed with randomly selected generalization sets. This ensures a fair comparison.

#### SVM agents

In case of SVM agents, a 10-fold cross validation is performed to optimize the parameters gamma and cost, using the build-in functions of the libSVM library. Finally, the SVMs were trained on the whole training set. In case of k-fold cross validation experiments, the data corresponding to the current fold are excluded from the parameter optimization as well as from the training.

### Data sets

We distinguish data sets for (i) the SSF compilation, (ii) the stability change prediction, and (iii) the disulfide bridge prediction. An overview of the validation tests is given in Table 1.

**Table 1:** **Overview of the validation data sets used in this work**

**Data set**	**Size**	**Prediction**	**Validation**	**Source/Ref.**
SP1	2648 mutations	*Δ* *Δ*G	5-fold cross validation	[12]
SP2	350 mutations	*Δ* *Δ*G	Performance test	[12]
SP3	1925 mutations	*Δ* *Δ*G	20-fold cross validation	[5,7]
SP4	1765 mutations	*Δ* *Δ*G	10-fold cross validation	ProTherm
MP	479 multi-point mutations	*Δ* *Δ*G	10-fold cross validation	ProTherm
SS1	75 disulfide bonds	S-S bond	Performance test	[32]
SS2	15 engineered disulfide bonds	S-S bond	Performance test	[32]

#### Statistical scoring functions compilation

For SSF compilation, a predefined list of PDB structure provided by PISCES service [30] was used. The set includes only structures with a resolution of 1.8 *Å* or better, an R-factor of 0.25 or better and a sequence identity of less than 25*%*. We excluded the following structures: (i) structures with incomplete backbones, (ii) structures found via a PDB search with the keywords virus or membrane, and (iii) structures with sequence similarity to proteins in the machine learning training or test sets (see Table 1) as inferred by BLAST (E-Values cutoff 10.0). The final set consists of 1302 monomeric and 1812 multimeric protein structures.

#### Stability change prediction

Five different data sets (SP1, SP2, SP3, SP4, and MP) were chosen for the stability change prediction experiments. All data sets are derived from the ProTherm database [31], which provides experimental thermodynamic parameters of protein stability, including the change in Gibbs free energy (*Δ* *Δ*G) upon mutations.

The first set (SP1) published by Dehouck *et al.* [12], provides *Δ* *Δ*G values for a set of 2648 single point mutations in 131 globular proteins. The set is limited to values of mutations that destabilize the structures less than 5 kcal/mol. The set contains measurements for various pH values and temperatures, but with a preference to pH values close to 7 and temperatures close to 25°C. In accordance with previous studies of Dehouck *et al.* [12], Worth *et al.* [13] and Pires *et al.* [14], we used a subset (SP2) of 350 point mutants for comparisons with other methods, where the remaining 2298 point mutants were used as training set. A 5-fold cross validation experiment was performed with the whole SP1 set.

The SP3 set published by Masso *et al.* [7] is a set of 1925 stability change measurements in 55 different proteins. It is based on a set published by Capriotti *et al.* [5]. In contrast to the SP1 set, it contains multiple *Δ* *Δ*G values for some mutations. Thus the set contains only 1299 distinct mutations. As in the studies of Pires *et al.* [14] and Masso *et al.* [7] we used this set for 20-fold cross validation.

We then derived two new data sets (SP4, MP) from the ProTherm database. In total the sets provide 2244 distinct mutations and the corresponding change in free energy of unfolding in water (*Δ* *Δ*G_H20_). The SP4 set consists of 1765 single point mutants, while the MP set provides 479 mutants with multiple mutations. Both sets were restricted to entries with a pH value between 5.5 and 8.5. In cases where ProTherm provides multiple *Δ* *Δ*G values for a certain mutation we chose the entry experiment conditions closer to 25°C and a pH of 7. In case of entries with equal conditions, we chose the median value or in case of two entries the older one. We carefully checked all entries in the two data sets by consulting the original articles and corrected or removed erroneous data. Data sets SP4 and MP are listed in Additional files 2 and 3 respectively.

#### Disulfide bridge prediction

Two recently published data sets [32] were used to investigate the power of the disulfide bridge prediction. The first set, SS1, includes 75 single chain X-ray structures with a resolution of 1.5*Å* or higher. Furthermore, each of the structures contains exactly one disulfide bridge. For the prediction experiments the cysteine residues responsible for the disulfide bonds were exchanged to alanine by simply keeping the main chain and *C* ^*β*^ coordinates, removing the *S* ^*γ*^ and changing the residue type to ALA in the PDB file. We then relax the structures in a similar way as Salam and coworkers. We therefore added missing loops with MODELLER [33]. Subsequently, we performed an energy minimization using UCSF Chimera [34] with 5000 steps steepest descent and 1000 steps conjugate gradient. The second set, SS2, provides 13 proteins with 15 engineered disulfide bonds and represents a subset of the 24 proteins published by Dani *et al.* [35].

### Mutation scan

Besides the change in stability prediction of given point mutations, MAESTRO provides a scan mode for the most stabilizing or destabilizing combination of point mutations. Three different algorithms are implemented for this task: (i) an optimal search, (ii) a greedy search, and (iii) an evolutionary algorithm. For all three algorithms mutation constraints, like the allowed substitutions or the number of mutation points, can be defined.

In case of an optimal search, all possible combinations of point mutations, which fit the given constraints, are calculated. This approach guarantees optimal results, but it is possibly very time-consuming, depending on the size of the protein and the given constraints.

The second algorithm, greedy search, is an iterative approach, where in each iteration a mutant is extended by the most stabilizing or destabilizing point mutation. This algorithm is fast, but cannot guarantee an optimal search result.

The evolutionary algorithm (EA) is implemented as a multi-population system with fitness driven parent selection, crossing over, point mutations and migration between the populations. The parameters mutation rate, migration rate and population size have been optimized.

### Disulfide bond scan

The disulfide bond scan is similar to the optimal mutation scan. First, all residue pairs with a *C* ^*β*^- *C* ^*β*^ distance within 5 *Å* are considered as potential binding partners. Then all possible pairs are subsequently mutated to cysteine and the mutants are rated by a score called *S*_*ss*_. This score includes three components, the predicted stability change *Δ* *Δ*G and two penalties which describe the geometric setup in the site considered for mutation.

The penalties are calculated from a distance distribution analysis of *C* ^*α*^ and *C* ^*β*^ atoms of disulfide bonded cysteines in 20711 PDB structures. The data set is derived from the PISCES service [30] and includes structures with a resolution of 2.5 *Å* or better and a sequence identity of less than 60*%*. The first penalty is $\phantom {\dot {i}\!}P_{\beta } = 1 - f(d_{C^{\beta } C^{\beta }})$, where *f*(*d*) is the relative frequency of the occurrence of a certain *C* ^*β*^- *C* ^*β*^ distance. The second penalty $\phantom {\dot {i}\!}P_{\alpha \beta } = 1 - f(d_{C^{\alpha } C^{\alpha }} - d_{C^{\beta } C^{\beta }})$ describes the differences between the *C* ^*α*^- *C* ^*α*^ and *C* ^*β*^- *C* ^*β*^ distances respectively. For the final disulfide bond score *S*_*ss*_, the predicted values for *Δ* *Δ*G, *P*_*β*_ and *P*_*αβ*_ are transformed to their respective z-scores for all potential binding partners:

(9) $$ S_{ss} = \frac{Z_{\Delta\Delta G} + Z_{P_{\beta}} + Z_{P_{\alpha\beta}}}{\sqrt{3}}  $$

The potential binding partners are ranked by *S*_*ss*_.

## Results and discussion

We conducted a series of experiments to test the performance of MAESTRO and to compare it with other state of the art methods. First, we show the predictive power of our approach on protein stability data and we analyze the influence of the three types of predictions agents on the consensus prediction. We then explore the pros and cons of the three different mutation scan methods. Finally, we assess the adequacy on the prediction of disulfide bonds.

In addition to the *Δ* *Δ*G prediction, MAESTRO provides a *Δ*Score value, based on the SSFs described before. Below, results labeled with MAESTRO-Score correspond to the *Δ*Score values, while results labeled with MAESTRO correspond to the *Δ* *Δ*G predictions.

The meaning of the sign of a *Δ* *Δ*G varies from data set to data set. We defined negative *Δ* *Δ*G as an increase in the stability of a protein, while positive values indicates a destabilization. For the following analyses we adapted all data sets to this definition.

### Stability change prediction upon mutations

Five different stability data sets were used (see Table 1). Four of these sets provide data on single point mutations (SP1, SP2, SP3, and SP4). The MP set provides data on multi-point mutations. Detailed per mutation results are listed in Additional file 4.

#### Single point mutations

**Figure 2:**
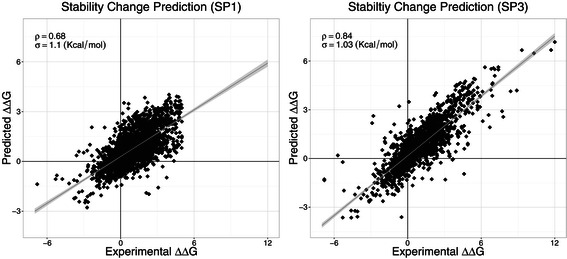
Correlation with experimental data. Regression results for MAESTRO on the single mutation data sets SP1 (left) and SP3 (right).

We first compare the performance based on the largest data set (SP1) and the associated subset (SP2). In the first experiment, a 5-fold cross validation on the SP1 set was performed. MAESTRO achieved a Pearson’s correlation coefficient of *ρ*=0.68 with standard error of *σ*=1.10 kcal/mol (see Figure 2), compared with a correlation of *ρ*=0.69 with *σ*=1.06 reported by Pires *et al.* [14] and *ρ*=0.63 with *σ*=1.15 reported by Dehouck *et al.* [12].

Several authors use the SP2 subset of 350 Mutants to compare their method for stability change prediction. Thus we also performed a benchmark on SP2 and trained MAESTRO on the remaining 2298 mutations in the SP1 set. It has to be noted that some of methods failed to compute some mutations, resulting in a subset of 309 predictions in common. Additionally, mutations with a high impact on the protein stability are frequently of special interest. Therefore, a second subset of SP2 consisting of mutations with |*Δ* *Δ*G|≥2 kcal/mol is also considered. Results are shown in Table 2. In summary, MAESTRO yields a better correlation than most of the competitors. Besides the good results of our *Δ* *Δ*G prediction, also the *Δ*Scores provide a reasonable performance, which is even more remarkable since the underlying SSFs are not specifically trained on a certain SP data set.

**Table 2:** **Performance comparison using the SP2 set**

**Method**	**#Predictions** ^**a**^	**Pearson’s** ***ρ*** ^**b**^	***σ*** ** (kcal/mol)** ^**b**^
AUTOMUTE	315	0.46/0.45/0.45	1.43/1.46/1.99
CUPSAT	346	0.37/0.35/0.50	1.91/1.96/2.14
Dmutant	350	0.48/0.47/0.57	1.81/1.87/2.31
Eris	334	0.35/0.34/0.49	4.12/4.28/3.91
I-Mutant-2.0	346	0.29/0.27/0.27	1.65/1.69/2.39
PopMuSiC-2.0	350	0.67/0.67/0.71	1.16/1.19/1.67
SDM	350	0.52/0.53/0.63	1.80/1.81/2.11
mCSM	350	0.73/0.74/0.82	1.08/1.10/1.48
MAESTRO-Score	350	0.56/0.57/0.68	−/ −/ −
MAESTRO	350	0.70/0.69/0.76	1.13/1.17/1.67

Results except for MAESTRO are taken from Dehouck *et al.* [12] and Pires *et al.* [14] respectively. ^a^The test set contains 350 entries, however several methods failed to compute the *Δ* *Δ*G prediction for some mutants, resulting in a reduced number of predictions. In these cases *Δ* *Δ*G was set to 0.0 kcal/mol for calculating the correlation coefficient. ^b^Three values are given for Pearson’s *ρ* as well as for the associated standard errors. They correspond (i) to the whole validation set, (ii) the subset of 309 mutants for which all methods provide a result, and (iii) the subset of 87 mutants with an experimental *Δ* *Δ*G≥2 kcal/mol or *Δ* *Δ*G≤2 kcal/mol respectively.

We performed a 20-fold cross validation experiment on the third widely used data set SP3. MAESTRO achieved a *ρ*=0.84 with *σ*=1.03 on this set (see Figure 2). In comparison to *ρ*=0.79 with *σ*=1.10 reported by Masso *et al.* [7] and *ρ*=0.82 with *σ*=1.00 reported by Pires *et al.* [14].

As mentioned before, we introduced a new data set on single point mutations (SP4). In a 10-fold cross validation experiment, MAESTRO achieved a *ρ*=0.68 with *σ*=1.31 on this set. Therewith the results on this set are similar to the results on the other single point sets.

**Figure 3:**
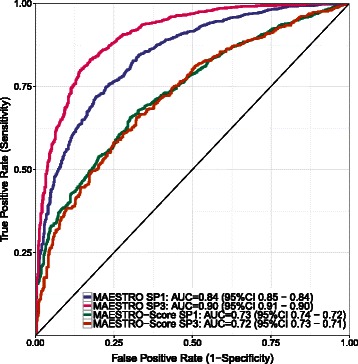
Binary classification. Classification performance of MAESTRO-Score and MAESTRO on the data sets SP1 and SP2. The data are derived from n-fold cross validation experiments.

In addition to the regression experiments, we performed classification tests. MAESTRO achieved an accuracy of 0.82 on the SP1 set, an accuracy of 0.84 on the SP3 set and an accuracy of 0.83 on the new SP4 set. ROC curves and AUC values for the data sets SP1 and SP3 are provided in Figure 3. Detailed results and a comparison with other methods are shown in Table S1 (Additional file 1).

Similar to the work of Pires *et al.* [14] we performed several blind tests to investigate the generalization qualities of our approach, when (i) a certain mutation site, (ii) a certain wild type amino acid, (iii) a certain exchange amino acid type and (iv) a certain protein is never used in the training.

A comparison with the method mCSM on the cases (i) and (iv) is given in Table 3. In both cases the loss in the prediction power is considerably smaller than reported by Pires *et al.* [14]. Further results are given in Tables S2, S3 and S4 (Additional file 1). In all tests for case (i), (ii) and (iii) the performance decreases marginally. Only case (iv) shows a notable performance decrease, indicating that there is some overfitting in terms of structural features. In sum, on all single point mutation data sets, MAESTRO is competitive to the leading prediction services.

**Table 3:** **Performance comparison on blind tests**

**Method**	**Data set**	**Validation**	**Pearson’s** ***ρ***	***σ*** ** (kcal/mol)**
mCSM^a^	SP1	5-fold Position^c^	0.54	1.23
MAESTRO	SP1	5-fold Position^c^	0.67	1.12
mCSM^a^	SP1	5-fold Protein^c^	0.51	1.26
MAESTRO	SP1	5-fold Protein^d^	0.63	1.17
mCSM^a^	SP1 351^d^	Blind Position	0.67	1.19
DUET^b^	SP1 351^d^	Blind Position	0.71	1.13
MAESTRO	SP1 351^e^	Blind Position	0.71	1.16

^a^Results derived from Pires *et al.* [14], supplementary material. ^b^Results derived from Pires *et al.* [15]. ^c^5-fold cross validation on position level. All mutations of a certain mutation site are either in the test or training set. ^d^5-fold cross validation on protein level. All mutations of a certain protein are either in the test or training set. ^e^Blind test on a subset of the SP1 data set, provided by Pires *et al.* [14]. The set includes 351 mutants, whose positions are not in the remaining training set.

#### Multi-point mutations

To our knowledge, none of the competitive services listed in Table 2 provides a stability change prediction on multi-point mutations. Tian *et al.* [36] report the prediction software Prethermut for multi-point mutations, as well as a training set derived from the ProTherm database and results for a 10-fold cross validation experiment on that set. From our point of view, this training set has some serious quality issues: First, the authors did not check the data derived from the ProTherm database for erroneous inputs, as recommended at the ProTherm website. We checked the top ten stabilizing and the top ten destabilizing mutations as well as a random sample of 100 mutants of the data set. In these samples we found eight entries which are listed with wrong sign for *Δ* *Δ*G. In the worst case the difference between *Δ* *Δ*G in the data set and the published *Δ* *Δ*G is 27.4 (see Table S10, Additional file 1). Second, the authors did not strictly follow their own rules they described for treating multiple ProTherm entries for the same mutation. Therefore, no objective comparison with Prethermut could be performed.

We assessed the performance of MAESTRO on our own sets in two 10-fold cross validation experiments. In the first experiment we joined the SP4 and MP set, to test how well MAESTRO performs on multi-point mutations, if it is trained on a mixed set of single and multi-point mutations. In this experiment our method achieved a Pearson’s correlation coefficient of *ρ*=0.71 with a standard error of *σ*=1.52 for multi-point mutations, and a *ρ*=0.69 with *σ*=1.36 on the whole mixed set. In the second experiment we performed the 10-fold cross validation on the MP set, which only includes multi-point mutations. Here, MAESTRO achieves a *ρ*=0.77 with *σ*=1.41 for multi-point mutations. In Table 4 the performance on different numbers of mutations is shown. While the correlation increases significantly, the classification accuracy on the MP set increases only slightly from 0.89 to 0.90, in case of a separated training.

**Table 4:** **10-fold cross validation results for our own data sets SP4 (single point) and MP (multi-point), as well on a joined data set which include the mutations of SP4 and MP**

**Number of mutations**	**Number of entries**	**SP4 set**		**MP set**		**Joined set**	
		***ρ***	***σ***	***ρ***	***σ***	***ρ***	***σ***
1	1765	0.68	1.31			0.68	1.32
>1	479			0.77	1.41	0.71	1.52
2	285			0.70	1.56	0.64	1.69
3	109			0.84	1.06	0.80	1.14
≥4	85			0.88	1.27	0.84	1.37
≥1	2244					0.69	1.36

These results indicate, that the prediction power can be improved if the training is performed on separated data sets for single point and multi-point mutants respectively. Overall, the predictive power on multi-point mutations is slightly better than on single point mutations. This outcome implies that MAESTRO can be used to scan a protein for multi-point (de)stabilizing mutants *per se*.

### Mutation scan

We use the term *n*-point mutation below to indicate the final number of sites where a substitution was introduced. The number of allowed sites exposed to mutations *a* is either simply the sequence length or a subset of residues. For the purpose of scanning for *n*-point mutation three methods were implemented, an optimal search, a greedy search and an evolutionary algorithm (EA).

As the number of combinations is growing exponentially with the number of allowed sites, the optimal search is potentially very time consuming. Thus experiments with the optimal search were limited to 3-point mutations and *a*≤30. This results in a maximum of 2.8·10^7^ combinations. The other methods were also tested with a larger number of allowed sites. For the test set we randomly selected eight protein structures of any size and two structures with exactly 30 residues from the PDB database. Additional selection criteria were: (i) The structure was resolved by X-ray with a resolution of 2*Å* or better. (ii) The structure may not contain DNA or RNA. (iii) Virus and membrane proteins were excluded.

In case of a small number of allowed mutation sites (*a*≤30), the EA search was able to find the optimal result in all test cases, the greedy search failed in two cases. Further experiments with the greedy and EA search were performed, without restrictions in the allowed mutation sites and *n*=3, 5, or 10. In these experiments the EA search performs better than the greedy search. Detailed results are shown in Table S9 (Additional file 1).

### Confidence estimation

**Figure 4:**
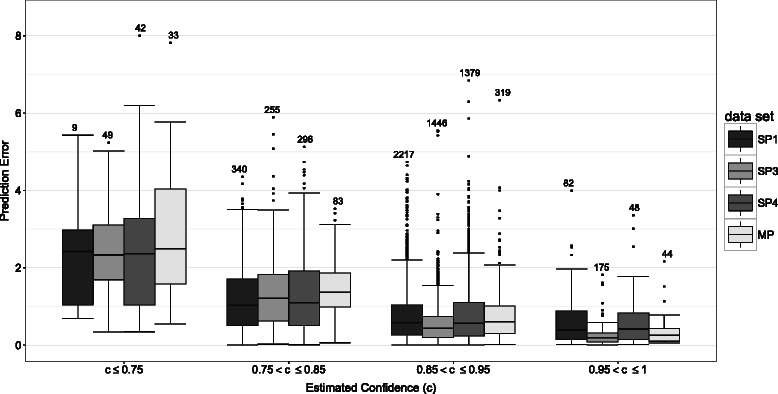
Confidence estimation and prediction error. Deviation between experimental determined *Δ* *Δ*G values and the predictions for different confidence value ranges. The prediction error is defined as the absolute difference between the experimental determined *Δ* *Δ*G and the predicted *Δ* *Δ*G. Data are given for the three main single point mutation sets (SP1, SP3, SP4) as well as the multi-point mutation set (MP). The numbers of prediction per group are shown at the top. In all cases, the deviation shrinks with higher confidence values.

As described before, MAESTRO provides a confidence estimation for a prediction, which is based on the standard deviation of the single agent predictions. As shown in Figure 4, the deviation between experimentally determined and predicted *Δ* *Δ*G values and therewith the standard error decreases with higher confidence values.

Especially in combination with a mutation scan, the confidence estimation is a simple but effective tool to assess a prediction.

### Prediction agents

The basic idea behind a multi-agent prediction was to improve the prediction power in relation to a single machine learning method and on the other hand to limit the risk of outliers and overfitting. We tested the prediction power of each agent, based on the n-fold cross validation experiment as well as the performance test based on the SP2 set, shown before.

We performed ten runs for each data set and report average, minimum and maximum for correlation coefficient and standard error (Table S5, Additional file 1). Depending on the data set and the run either the NN agents or the SVM agents performed better. The MLR agents performs nearly equally good on each data set, except on the MP set. Without the specialized agents, the performance decreases considerably. In some cases, the NN or SVM agents alone would even perform better than the combined *Δ* *Δ*G prediction. However, the ANN and SVM have larger variations in *ρ* than the combined score in the different runs. The ranges for *ρ* are given in Table S5 in Additional file 1. Overall, by integrating various predictors the method gains robustness compared to the single agents.

Utilizing the three different methods, ANN, SVM and MLR in combination also increases the efficiency of the confidence estimation. We performed an experiment using an ensemble of seven ANNs instead of the seven MAESTRO agents for deriving the confidence estimation. In comparison to MAESTRO results (Figure 4), this approach exhibits a less pronounced relationship between high confidence values and correct predictions, as shown in Figure S2 in Additional file 1.

The final version of MAESTRO was trained on set SP4 and MP. Alternative configurations trained on SP1 and SP3 are available on our web pages.

### Disulfide bond prediction

MAESTRO provides a special mode for the prediction of suitable disulfide bonds to stabilize a protein structure. In two experiments we show the performance of our approach on this task. For both cases, we trained MAESTRO on the single point data sets (SP1, SP3, and SP4). The best performance was obtained with set SP3. The corresponding results are reported below and in Additional file 1.

The first experiment was performed on the SS1 set, where the cysteine pairs in disulfide bonds were mutated to alanine. The goal in this experiment was to find the original binding partners out of all possible binding candidates. As mentioned before, the set of possible binding partners include all residue pairs in a structure with a *C* ^*β*^- *C* ^*β*^ distance within 5 *Å*. We performed the test on both, the original PDB structures, where only the residue type was changed from cysteine to alanine while keeping the coordinates as in the PDB file, and on the relaxed structures. The relative rank given below is the absolute rank of the considered native bond divided by the number of possible binding partners. For the unchanged PDB structures the average/median relative rank of MAESTRO is 0.08/0.06 compared to 0.06/0.03 of the method of Salam *et al.* [32]. The corresponding absolute average/median ranks for MAESTRO are 7.2/5.0 (Salam *et al.* [32] does not provide absolute ranks). In 13 cases MAESTRO ranked the native bond on top compared to 16 cases of the method of Salam *et al*. From the relaxed structures of test set SS1, in two of the 75 proteins the *C* ^*β*^- *C* ^*β*^ distance of the mutated cysteines is larger than 5*Å*. For the remaining 73 proteins, in 25 cases the relative rank decreases, in 48 it increases. In six cases MAESTRO ranked the native bond on top. The relative average/median rank increases to 0.13/0.08 (absolute rank: 13.5/8). The major reason for the increase is that after loop modelling and minimization additional alternative positions with a *C* ^*β*^- *C* ^*β*^ within the cutoff 5*Å* appear. The scores and geometric penalties for the native cysteine pair positions are to an almost equal amount better or worse in the relaxed structures. Detailed results are provided in Table S7 (Additional file 1). When comparing our results to those of Salam *et al.* it has to be considered, that Salam *et al.* used this set in a cross validation experiment. In contrast, for MAESTRO these 75 proteins where not included the training data.

Energy minimization after introducing the cysteine to alanine mutation allows the structures to relax towards a conformation which is not constrained by the disulfide bridge. To which extend these models resemble the native fold is debatable. Moreover, it is unclear whether these proteins can adopt a stable fold without the disulfide bonds.

The second experiment was performed on the SS2 set comprising 13 proteins without disulfide bonds in the wild type, where variants with stabilizing disulfide bonds have been engineered. As shown in Table 5, our method reaches an average/median relative rank of 0.20/0.16, whereas Salam *et al.* [32] reported an average/median rank of 0.31/0.23 for this task. The corresponding absolute average/median ranks are 24/11 and 31/19 respectively. In addition, we evaluated the SS-bond scoring components separately, *Δ* *Δ*G only, score only and geometrics penalty only. For *Δ* *Δ*G the average/median ranks increase to 0.32/0.30 (absolute 35/30), for the score only to 0.38/0.32 (absolute 42/40) and for the geometric penalty only to 0.29/0.23 (absolute 35/17). Adding the geometric penalty to the *Δ* *Δ*G and energy based score respectively improves the performance considerably. Interestingly, the geometric penalty alone performs as good as the method of Salam *et al.* [32].

**Table 5:** **Prediction of disulfide bonds in 15 structures with known engineered disulfide bonds**

**PDB ID**	**Mutation** ^**a**^	**MAESTRO**		**MAESTRO-Score**		**Salam** ***et al.***	
		**Abs.Rank**	**Rel.Rank**	**Abs.Rank**	**Rel.Rank**	**Abs.Rank**	**Rel.Rank**
1FG9	Glu7:A–Ser69:A	1	0.04	0	0.00	15	0.71
1LMB	Tyr88:3–Tyr88:4	10	0.20	9	0.18	32	0.48
1RNB	Ala43–Ser80	2	0.03	9	0.14	3	0.07
1RNB	Ser85–His102	33	0.52	40	0.63	0	0.00
1SNO	Gly79–Asn118	3	0.04	4	0.05	22	0.34
1XNB	Ser100–Asn148	1	0.01	13	0.10	8	0.08
2CBA	Leu60–Ser173	66	0.45	68	0.46	55	0.47
2CI2	Thr22–Val82	5	0.18	4	0.14	0	0.00
2LZM	Ile9–Leu164	31	0.44	38	0.54	13	0.21
2RN2	Cys13–Asn44	14	0.16	21	0.24	25	0.33
2ST1	Thr22–Ser87	37	0.16	30	0.13	44	0.23
3GLY	Asn20–Ala27	104	0.39	163	0.62	187	0.82
3GLY	Thr246–Cys320	35	0.13	34	0.13	19	0.08
4DFR	Pro39–Cys85	11	0.10	11	0.10	16	0.13
9RAT	Ala4–Val118	8	0.15	10	0.19	25	0.68
**Average**		**2** **4**	**0** **.** **2** **0**	**3** **0**	**0** **.** **2** **4**	**3** **1**	**0** **.** **3** **1**
**Median**		**1** **1**	**0** **.** **1** **6**	**1** **3**	**0** **.** **1** **4**	**1** **9**	**0** **.** **2** **3**

Abs.Rank: absolute rank, Rel.Rank: relative rank. ^a^The letter or digit after the colon denotes the chain(s) used in case of multi-chain PDB entries.

These experiments on SS2 resemble the real application of the predictor, to provide a list of potentially stabilizing SS-bonds for a given protein of known structure. However, disulfide bonds introduced on other positions might result in as good or even better stability. It would be certainly interesting to have experimental data on the top ranking prediction for each of the test proteins.

MAESTRO provides competitive results on this prediction task. In opposite to Salam *et al.*, our method is not specially trained on disulfide bond data. Therewith, MAESTRO cannot possibly overfit on this task. Further, MAESTRO performs no minimization or other manipulation of the structure for its prediction but simply operates on the plain PDB file. No third party software is required and the run time is just in the order of a few seconds to minutes. In the experiments on the data sets SS1 and SS2, the average run time per structure was about 3 seconds, but not longer than 25 seconds, on a standard desktop PC.

## Conclusions

MAESTRO is a freely available versatile tool in the field of stability change prediction upon point mutations. The performance is comparable to mCSM, our main competitor method. MAESTRO offers additional features, which extends its range of applicability compared to other methods. First, it performs predictions on multi-point mutations. Second, it allows massive scans for stabilizing and destabilizing mutants under given restrictions such as amino acid types or solvent accessibility. Third, it utilizes a special mode for stabilizing disulfide bond prediction. The performance thereof is comparable to the recently published method of Salam and coworkers.

Like all structure based prediction methods MAESTRO’s applicability is limited by the availability of a reliable 3d structure. Luckily, the PDB database increases rapidly in size delivering more and more suitable input. For the competitor method PoPMuSiC it has been shown that also structures created by comparative modeling can be sufficient for stability prediction [18]. To which extent this is also true for MAESTRO is still an open question and shall be investigated in a future study.

A second limitation of MAESTRO, as of all machine learning approaches, is the limited amount of experimental data for training and validation. ProTherm provides a relatively large data set with some caveats which can be resolved by a careful selection protocol. However, many data are derived from a few model proteins which are often used for biochemical and biophysical investigations. Thus, the content of ProTherm may not be considered as representative, and in comparison to PDB, ProTherm is growing rather slow. This last point is possibly also the reason for the observed overfitting on the wild type structure. If a certain protein was not present in the training at all, the average performance decreased.

All leading methods reach correlation coefficients between 0.7 and 0.8 on the different data sets. One can speculate that currently there is not more (precise) information in the training data which can be pulled out by machine learning approaches. A solely energy based approach may help to overcome this problem. It is certainly desirable to develop better energetic models.

Especially for the disulfide bond prediction but also to some extent for the single point mutation we observed some complementarity between different methods. So probably a meta method might further improve the prediction accuracy. At least, for users of stability prediction software it is recommended to consult different services to gain confidence.

## Additional files

Additional file 1
**Additional Results.** Additional results on blind tests, disulfide bond predictions and data set problems.

Additional file 2
**SP4 Data Set.** Single point mutation data set SP4 derived from ProTherm.

Additional file 3
**MP Data Set.** Multiple point mutation data set MP derived from ProTherm.

Additional file 4
**N-fold Cross Validation Results.** Per-mutation results for the n-fold cross validation experiments.

## References

[1] Stefl S, Nishi H, Petukh M, Panchenko AR, Alexov E (2013). Molecular mechanisms of disease-causing missense mutations. J Mol Biol..

[2] Cobb RE, Sun N, Zhao H (2013). Directed evolution as a powerful synthetic biology tool. Methods.

[3] Teng S, Srivastava AK, Wang L (2010). Sequence feature-based prediction of protein stability changes upon amino acid substitutions. BMC Genomics.

[4] Huang L-T, Gromiha MM, Ho S-Y (2007). iPTREE-STAB: interpretable decision tree based method for predicting protein stability changes upon mutations. Bioinformatics.

[5] Capriotti E, Fariselli P, Casadio R (2005). I-Mutant2.0: predicting stability changes upon mutation from the protein sequence or structure. Nucleic Acids Res..

[6] Cheng J, Randall A, Baldi P (2006). Prediction of protein stability changes for single-site mutations using support vector machines. Proteins.

[7] Masso M, Vaisman II (2008). Accurate prediction of stability changes in protein mutants by combining machine learning with structure based computational mutagenesis. Bioinformatics.

[8] Parthiban V, Gromiha MM, Schomburg D (2006). CUPSAT: prediction of protein stability upon point mutations. Nucleic Acids Res..

[9] Zhou H, Zhou Y (2002). Distance-scaled, finite ideal-gas reference state improves structure-derived potentials of mean force for structure selection and stability prediction. Protein Sci..

[10] Guerois R, Nielsen JE, Serrano L (2002). Predicting changes in the stability of proteins and protein complexes: a study of more than 1000 mutations. J Mol Biol..

[11] Yin S, Ding F, Dokholyan NV (2007). Eris: an automated estimator of protein stability. Nat Methods.

[12] Dehouck Y, Grosfils A, Folch B, Gilis D, Bogaerts P, Rooman M (2009). Fast and accurate predictions of protein stability changes upon mutations using statistical potentials and neural networks: PoPMuSiC-2.0. Bioinformatics.

[13] Worth CL, Preissner R, Blundell TL (2011). SDM–a server for predicting effects of mutations on protein stability and malfunction. Nucleic Acids Res..

[14] Pires DEV, Ascher DB, Blundell TL (2014). mCSM: predicting the effects of mutations in proteins using graph-based signatures. Bioinformatics.

[15] Pires DEV, Ascher DB, Blundell TL (2014). DUET: a server for predicting effects of mutations on protein stability using an integrated computational approach. Nucleic Acids Res..

[16] Khan S, Vihinen M (2010). Performance of protein stability predictors. Hum Mutat..

[17] Potapov V, Cohen M, Schreiber G (2009). Assessing computational methods for predicting protein stability upon mutation: good on average but not in the details. Protein Eng Des Sel..

[18] Gonnelli G, Rooman M, Dehouck Y (2012). Structure-based mutant stability predictions on proteins of unknown structure. J Biotechnol..

[19] Gromiha MM, Sarai A (2010). Thermodynamic database for proteins: features and applications. Methods Mol Biol..

[20] Rokach L (2010). Ensemble-based classifiers. Artif Intelligence Rev..

[21] Sippl MJ (1993). Recognition of errors in three-dimensional structures of proteins. Proteins.

[22] Sippl MJ (1993). Boltzmann’s principle, knowledge-based mean fields and protein folding. An approach to the computational determination of protein structures. J Comput Aided Mol Des..

[23] Wiederstein M, Sippl MJ (2005). Protein sequence randomization: efficient estimation of protein stability using knowledge-based potentials. J Mol Biol..

[24] Lehmann EL, D’Abrera HJM. Nonparametrics: Statistical Methods Based on Ranks: Springer; 2006.

[25] Park SY, Yoo M-J, Shin J, Cho K-H (2011). SABA (secondary structure assignment program based on only alpha carbons): a novel pseudo center geometrical criterion for accurate assignment of protein secondary structures. BMB Rep..

[26] Voss NR, Gerstein M (2005). Calculation of standard atomic volumes for RNA and comparison with proteins: RNA is packed more tightly. J Mol Biol..

[27] Lopez R. Open NN: An Open Source Neural Networks C++ Library. 2014. http://www.cimne.com/flood. Accessed 7 Apr 2015.

[28] Chih-Chung C, Chih-Jen L (2011). LIBSVM: A Library for Support Vector Machines. ACM Trans Intell Syst Technol..

[29] Gallassi M, Davies J, Theiler J, Gough B, Jungman G, Alken P (2009). GNU Scientific Library Reference Manual, 3rd edn..

[30] Wang G, Dunbrack RL (2003). PISCES: a protein sequence culling server. Bioinformatics.

[31] Kumar MDS, Bava KA, Gromiha MM, Prabakaran P, Kitajima K, Uedaira H (2006). ProTherm and ProNIT: thermodynamic databases for proteins and protein-nucleic acid interactions. Nucleic Acids Res..

[32] Salam NK, Adzhigirey M, Sherman W, Pearlman DA (2014). Structure-based approach to the prediction of disulfide bonds in proteins. Protein Eng Des Sel..

[33] Sali A, Blundell TL (1993). Comparative protein modelling by satisfaction of spatial restraints. J Mol Biol..

[34] Pettersen EF, Goddard TD, Huang CC, Couch GS, Greenblatt DM, Meng EC (2004). UCSF Chimera–a visualization system for exploratory research and analysis. J Comput Chem..

[35] Dani VS, Ramakrishnan C, Varadarajan R (2003). MODIP revisited: re-evaluation and refinement of an automated procedure for modeling of disulfide bonds in proteins. Protein Eng..

[36] Tian J, Wu N, Chu X, Fan Y (2010). Predicting changes in protein thermostability brought about by single- or multi-site mutations,. BMC Bioinf..

