# Supplementary material for: MAESTRO - multi agent stability prediction upon point mutations
Source: BMC Bioinformatics. 2015 Apr 16;16:116. doi: 10.1186/s12859-015-0548-6 (PMC4403899; doi:10.1186/s12859-015-0548-6)
Supplement: Additional file 1 — Additional Results. Additional results on blind tests, disulfide bond predictions and data set problems. [file 12859_2015_548_MOESM1_ESM.pdf]

# MAESTRO - Additional Results

Josef Laimer, Heidi Hofer, Marko Fritz, Stefan Wegenkittl and Peter Lackner

February 13, 2015

## Contents

**1 Prediction Workflow** **2**

**2 Classification Performance** **3**

**3 Blind Tests** **3**

**4 Agents and SSF Performance** **6**

**5 Disulfide Bond Prediction** **9**

**6 Mutation Scan** **11**

**7 Misclassified ProTherm Entries** **13**

**8 Statistical Scoring Function Parameters** **13**

# 1 Prediction Workflow

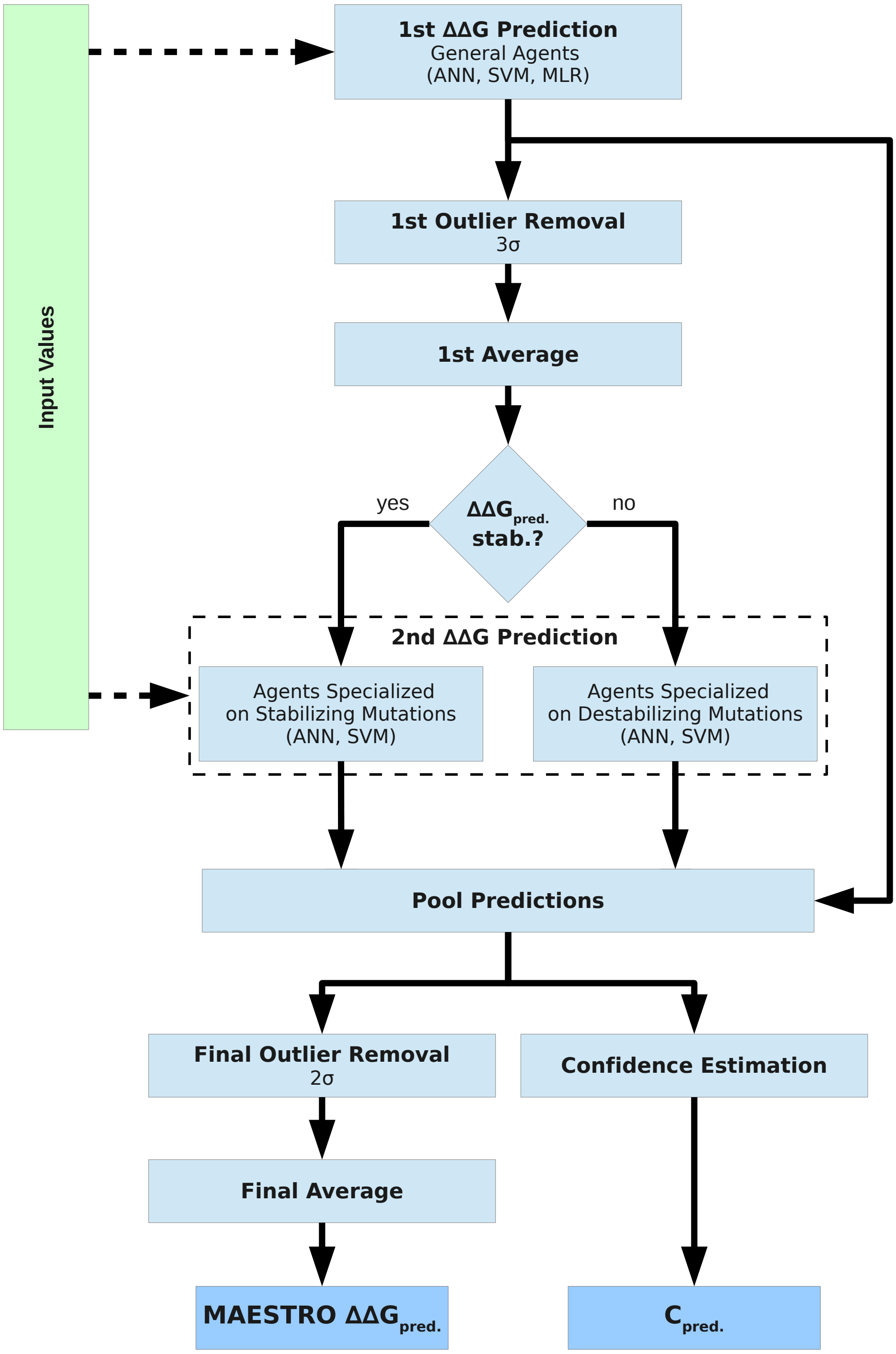


Figure S1: Prediction workflow. Depending on the data set between 2% and 8% of all agent predictions are excluded in the final outlier removal.

# 2 Classification Performance

Table S1 summarizes the performance on binary classification. Note that MAESTRO was not specially trained for binary classification, in contrast to the other tools listed in Table S1. Nevertheless, MAESTRO performs similar to the main competitor methods. A prediction is considered to be true positive or true negative, respectively, if the sign of the predicted ΔΔG (or score in case of MAESTRO−Score) matches the sign of the experimental determined ΔΔG. The results are based on the n-fold cross validation experiments (SP1 with 5-fold, SP3 with 20-fold, SP4 with 10-fold) as presented in the main results.

| Data set | Method | Acc. | Recall [+][a] | Prec. [+][a] | Recall [-][b] | Prec. [-][b] | MCC | AUC |
|---|---|---|---|---|---|---|---|---|
| SP1 | MAESTRO-Score | 0.65 | 0.71 | 0.36 | 0.63 | 0.88 | 0.29 | 0.73 |
| | MAESTRO | 0.82 | 0.59 | 0.61 | 0.89 | 0.88 | 0.48 | 0.84 |
| SP4 | MAESTRO-Score | 0.63 | 0.66 | 0.30 | 0.62 | 0.88 | 0.22 | 0.68 |
| | MAESTRO | 0.83 | 0.41 | 0.59 | 0.93 | 0.87 | 0.40 | 0.80 |
| SP3 | AUTOMUTE (RF)[c] | 0.86 | 0.70 | 0.81 | 0.93 | 0.88 | 0.66 | 0.91 |
| | I-Mutant 2.0[c] | 0.80 | 0.56 | 0.73 | 0.91 | 0.83 | 0.51 | - |
| | mCSM[c] | 0.86 | 0.67 | 0.82 | 0.94 | 0.87 | 0.65 | 0.90 |
| | MAESTRO-Score | 0.65 | 0.69 | 0.45 | 0.63 | 0.82 | 0.29 | 0.72 |
| | MAESTRO | 0.84 | 0.74 | 0.74 | 0.89 | 0.89 | 0.63 | 0.90 |

Table S1: Binary classification results for the SP1 and SP3 data sets. [a]Results for mutations that stabilize the structures. [b]Results for mutations with a destabilizing effect. [c]Data taken from Pires *et al.* (supplementary material) [5].

# 3 Blind Tests

All data sets used in this work contain multiple mutations for certain proteins or even certain mutation sites. In the experiments reported above, the possibly arising correlations introduced by this different types of mutations may eventually have led to a little overfitting on structure or position base. Thus, we performed blind tests to investigate the generalization capabilities of MAESTRO.

In the first experiments, the effect of the exclusion of certain **mutation sites** was investigated. We performed n-fold cross validation experiments, where all mutations of a mutation site are either exclusively in the training or in the test set. The n-fold cross validations were performed on the SP1 and the SP3 data set. Further, we show the performance on a low-redundancy subset derived from the SP1 data set, provided by Pires *et al.* [5]. The set includes 351 mutants. For this experiment MAESTRO was trained on the remaining 2297 mutations of the SP1. Regarding the results for this subset, Pires *et al.* remarked that *'It is important to point out that this data set may not be completely blind for PoPMuSiC, since the chosen mutations could have been considered while training its artificial neural network.'*

Table S2 shows that the prediction performance on the SP1 and SP4 data set only decreases marginally, in comparison to the 5-fold cross validation experiment ($\rho = 0.68$) and 10-fold cross validation experiment ($\rho = 0.68$), respectively, presented in the main results. In case of the blind test on the subset of 351 mutants the performance is similar to the results on the SP2 data set ($\rho = 0.70$). The relatively large difference in performance on the SP3 data set in comparison to the 20-fold cross validation experiment ($\rho = 0.84$) can be explained by the high number of mutations per site in this set[1].

[1] Average/median mutations per mutation site: SP1 . . . 1.85/1.00; SP3 . . . 3.05/2.00

| Method | Data set | Validation | Pearson's $\rho$ | $\sigma$ (kcal/mol) |
|---|---|---|---|---|
| mCSM[a] | SP1 | 5-fold cross validation | 0.54 | 1.23 |
| MAESTRO-Score | SP1 | 5-fold cross validation | 0.45 | - |
| MAESTRO | SP1 | 5-fold cross validation | 0.67 | 1.12 |
| | | | | |
| MAESTRO-Score | SP3 | 20-fold cross validation | 0.44 | - |
| MAESTRO | SP3 | 20-fold cross validation | 0.74 | 1.23 |
| MAESTRO-Score | SP4 | 10-fold cross validation | 0.40 | - |
| MAESTRO | SP4 | 10-fold cross validation | 0.65 | 1.36 |
| mCSM[a] | SP1 351 | blind test | 0.67 | 1.19 |
| PoPMuSiC[a] | SP1 351 | blind test | 0.73 | 1.09 |
| MAESTRO-Score | SP1 351 | blind test | 0.59 | – |
| MAESTRO | SP1 351 | blind test | 0.71 | 1.16 |

Table S2: Prediction performance in case of excluded mutation sites. [a]Data obtained from Pires *et al.* (supplementary material) [5].

In the second type of blind test experiments we investigated the effect of **excluded proteins**. This reflects best the real world application of a prediction method. Therefore we first performed n-fold cross validation experiments on the SP1, SP3 as well as on the SP4 data set, where all mutations of a certain protein are either exclusively in the training or in the test set. In a second set of experiments we aimed to determine the impact of sequence similarity between a protein in the training set and in the test set. All proteins in a certain set (SP1,SP3,SP4) were clustered by sequence similarity using BLASTclust with similarity cutoff of 30% identical residues in the alignment (BLASTclust parameter -S = 30, the remaining parameters were left at their default values). In the blind test a certain protein cluster is then either exclusively in the training or in the test set. We finally performed an experiment on data set SP1 where we used the n-fold definition as kindly provided by Pires *et al.* [5] on their web pages[2]. The results are summarized in Table S3 below.

| Method | Data set | Validation | Pearson's$\rho$ | $\sigma$ (kcal/mol) |
|---|---|---|---|---|
| mCSM[a] | SP1 | 5-fold cross validation | 0.51 | 1.26 |
| MAESTRO | SP1 | 5-fold cross validation | 0.63 | 1.17 |
| MAESTRO | SP1 | 5-fold cross validation (BLASTclust) | 0.63 | 1.17 |
| MAESTRO | SP1 | 5-fold cross validation (Pires def.) | 0.62 | 1.18 |
| MAESTRO | SP3 | 20-fold cross validation | 0.70 | 1.32 |
| MAESTRO | SP3 | 20-fold cross validation (BLASTclust) | 0.69 | 1.33 |
| MAESTRO | SP4 | 10-fold cross validation | 0.60 | 1.44 |
| MAESTRO | SP4 | 10-fold cross validation (BLASTclust) | 0.61 | 1.44 |

Table S3: Prediction performance in case of excluded proteins. [a]Data obtained from Pires *et al.* (supplementary material) [5].

In general, we observe a decrease in performance with this protein based blind test compared to the random n-fold tests (see results on single point mutations in the main text) and also compared to the blind test regarding the mutation site (Table S2). However, the performance decrease is less pronounced for MAESTRO then for mCSM. The appearance of homologous proteins in training set and test set has little impact on the results. The differently grouped 5-fold cross validation sets for data set S1 (ours vs. the mCSM ones) does not influence the MAESTRO result.

Besides the regression performance we analyzed the impact of the two blind test experiments on the binary classification performance. The results in Table S4 show that the classification performance is less affected as the regression performance.

[2]`http://bleoberis.bioc.cam.ac.uk/mcsm/data`

| Data set | Blind test | Acc. | Recall [+] | Prec. [+] | Recall [-] | Prec. [-] | MCC | AUC |
|---|---|---|---|---|---|---|---|---|
| SP1 | 5-fold mutation site | 0.81 | 0.55 | 0.59 | 0.88 | 0.87 | 0.45 | 0.83 |
| | 5-fold protein | 0.80 | 0.55 | 0.55 | 0.87 | 0.87 | 0.42 | 0.81 |
| | 5-fold protein (BLASTclust) | 0.80 | 0.53 | 0.55 | 0.87 | 0.86 | 0.41 | 0.81 |
| | 5-fold protein (Pires def.) | 0.79 | 0.56 | 0.54 | 0.86 | 0.87 | 0.42 | 0.81 |
| SP3 | 20-fold mutation site | 0.82 | 0.70 | 0.69 | 0.87 | 0.87 | 0.57 | 0.85 |
| | 20-fold protein | 0.81 | 0.70 | 0.67 | 0.85 | 0.87 | 0.55 | 0.85 |
| | 20-fold protein (BLASTclust) | 0.80 | 0.73 | 0.65 | 0.83 | 0.88 | 0.54 | 0.84 |
| SP4 | 10-fold mutation site | 0.82 | 0.39 | 0.57 | 0.93 | 0.86 | 0.37 | 0.79 |
| | 10-fold protein | 0.82 | 0.32 | 0.57 | 0.94 | 0.85 | 0.33 | 0.77 |
| | 10-fold protein (BLASTclust) | 0.82 | 0.39 | 0.57 | 0.93 | 0.86 | 0.37 | 0.78 |

Table S4: Classification performance on blind test experiments on mutation site and protein level.

Finally, we performed jack knife tests on the SP1 data set, where either a **wild type amino acid** or an **exchange amino acid** type was excluded from the training. In both cases the predictive power was reduced only marginally. The jack knife test on the wild type amino acids results in an overall $\rho = 0.65$ with $\sigma = 1.14$, while the jack knife test on the exchange amino acid results in an overall $\rho = 0.67$ with $\sigma = 1.13$.

# 4 Agents and SSF Performance

Here we present the performance of the SSFs (MAESTRO-Score) and the three agent types. The data shown in the following table, are the results of ten repeats for each experiment. In case of the n-fold validation experiments the folds were randomly defined for each repeat. For the blind-test experiment on the SP2 set (350 mutants), MAESTRO and therewith its agents were trained ten times on the remaining 2298 mutants of the SP1 set.

| **Data set** | **Validation** | **Agent Type** | **Pearson's $\rho$ avg.[min.,max.]** | **Spearman's $\rho$ avg.[min.,max.]** | **$\sigma$ (kcal/mol) avg.[min.,max.]** |
|---|---|---|---|---|---|
| SP1 | 5-fold | MAESTRO | 0.68 [0.67, 0.68] | 0.66 [0.65, 0.67] | 1.11 [1.10, 1.12] |
| | | MAESTRO No S.A. | 0.63 [0.62, 0.63] | 0.63 [0.62, 0.63] | 1.20 [1.19, 1.21] |
| | | NN Agents | 0.67 [0.63, 0.71] | 0.65 [0.60, 0.69] | 1.12 [1.05, 1.18] |
| | | SVM Agents | 0.68 [0.65, 0.72] | 0.67 [0.65, 0.71] | 1.09 [1.03, 1.11] |
| | | MLR Agents | 0.56 [0.56, 0.56] | 0.57 [0.57, 0.57] | 1.58 [1.58, 1.59] |
| | | MAESTRO-Score | 0.45 | 0.43 | - |
| SP2 | blind | MAESTRO | 0.69 [0.68, 0.70] | 0.65 [0.63, 0.67] | 1.15 [1.13, 1.17] |
| | | MAESTRO No S.A. | 0.67 [0.65, 0.68] | 0.63 [0.61, 0.65] | 1.18 [1.16, 1.21] |
| | | NN Agents | 0.66 [0.63, 0.69] | 0.62 [0.57, 0.66] | 1.20 [1.16, 1.24] |
| | | SVM Agents | 0.67 [0.65, 0.69] | 0.65 [0.63, 0.67] | 1.17 [1.15, 1.20] |
| | | MLR Agents | 0.61 [0.61, 0.61] | 0.57 [0.57, 0.57] | 1.53 [1.53, 1.53] |
| | | MAESTRO-Score | 0.56 | 0.49 | - |
| SP3 | 20-fold | MAESTRO | 0.83 [0.82, 0.84] | 0.80 [0.79, 0.81] | 1.05 [1.03, 1.08] |
| | | MAESTRO No S.A. | 0.76 [0.75, 0.77] | 0.75 [0.74, 0.76] | 1.23 [1.21, 1.25] |
| | | NN Agents | 0.82 [0.80, 0.84] | 0.79 [0.78, 0.81] | 1.04 [0.99, 1.09] |
| | | SVM Agents | 0.82 [0.78, 0.86] | 0.80 [0.76, 0.84] | 1.03 [0.93, 1.14] |
| | | MLR Agents | 0.56 [0.56, 0.56] | 0.58 [0.58, 0.58] | 1.77 [1.77, 1.77] |
| | | MAESTRO-Score | 0.44 | 0.43 | - |
| SP4 | 10-fold | MAESTRO | 0.68 [0.67, 0.68] | 0.64 [0.63, 0.65] | 1.33 [1.32, 1.33] |
| | | MAESTRO No S.A. | 0.61 [0.60, 0.62] | 0.59 [0.58, 0.60] | 1.47 [1.46, 1.49] |
| | | NN Agents | 0.69 [0.65, 0.71] | 0.65 [0.61, 0.67] | 1.29 [1.25, 1.38] |
| | | SVM Agents | 0.67 [0.66, 0.70] | 0.64 [0.63, 0.67] | 1.31 [1.26, 1.33] |
| | | MLR Agents | 0.49 [0.49, 0.49] | 0.49 [0.49, 0.49] | 2.03 [2.02, 2.03] |
| | | MAESTRO-Score | 0.40 | 0.38 | - |
| MP | 10-fold | MAESTRO | 0.75 [0.73, 0.77] | 0.69 [0.67, 0.70] | 1.45 [1.41, 1.51] |
| | | MAESTRO No S.A. | 0.66 [0.64, 0.67] | 0.64 [0.63, 0.66] | 1.65 [1.63, 1.68] |
| | | NN Agents | 0.77 [0.70, 0.79] | 0.71 [0.64, 0.73] | 1.36 [1.30, 1.52] |
| | | SVM Agents | 0.76 [0.74, 0.77] | 0.71 [0.69, 0.72] | 1.38 [1.35, 1.42] |
| | | MLR Agents | 0.46 [0.44, 0.46] | 0.42 [0.41, 0.44] | 2.34 [2.33, 2.35] |
| | | MAESTRO-Score | 0.32 | 0.27 | - |

Table S5: Agent type and SSF (MAESTRO-Score) performance on n-fold cross validation experiments and the SP2 data set, in comparison to the combined prediction (MAESTRO). MAESTRO No S.A. refers to an experiment where the specialized agents are disabled.

## Agents Classification Performance

In the following table we show the classification performance of the three agent types (NN, SVM and MLR) in comparison to the whole MAESTRO ensemble and MAESTRO with disabled specialized agents (MAESTRO No S.A.). The results are derived from the n-fold cross validation experiments on the SP1 set as well as on the blind test on SP2, as presented before and in the main text.

| Data set | Agent Type | Acc. | Recall [+][a] | Prec. [+][a] | Recall [-][b] | Prec. [-][b] | MCC | AUC |
|---|---|---|---|---|---|---|---|---|
| SP1 | MAESTRO | 0.82 | 0.59 | 0.61 | 0.89 | 0.88 | 0.48 | 0.84 |
| | MAESTRO No S.A. | 0.77 | 0.62 | 0.5 | 0.82 | 0.88 | 0.4 | 0.81 |
| | NN Agents | 0.82 | 0.51 | 0.64 | 0.91 | 0.86 | 0.46 | 0.85 |
| | SVM Agents | 0.83 | 0.43 | 0.69 | 0.94 | 0.85 | 0.45 | 0.83 |
| | MLR Agents | 0.63 | 0.85 | 0.36 | 0.56 | 0.93 | 0.35 | 0.77 |
| SP1 | MAESTRO | 0.80 | 0.53 | 0.55 | 0.87 | 0.86 | 0.41 | 0.81 |
| BLASTclust[c] | MAESTRO No S.A. | 0.74 | 0.56 | 0.44 | 0.79 | 0.86 | 0.32 | 0.76 |
| | NN Agents | 0.80 | 0.42 | 0.59 | 0.91 | 0.84 | 0.38 | 0.82 |
| | SVM Agents | 0.80 | 0.37 | 0.62 | 0.93 | 0.83 | 0.37 | 0.80 |
| | MLR Agents | 0.62 | 0.81 | 0.35 | 0.56 | 0.91 | 0.31 | 0.76 |
| SP1 | MAESTRO | 0.79 | 0.56 | 0.54 | 0.86 | 0.87 | 0.42 | 0.81 |
| Pires def.[d] | MAESTRO No S.A. | 0.74 | 0.58 | 0.44 | 0.78 | 0.87 | 0.34 | 0.77 |
| | NN Agents | 0.80 | 0.49 | 0.57 | 0.89 | 0.86 | 0.40 | 0.82 |
| | SVM Agents | 0.81 | 0.38 | 0.62 | 0.93 | 0.84 | 0.38 | 0.80 |
| | MLR Agents | 0.62 | 0.82 | 0.36 | 0.56 | 0.92 | 0.32 | 0.76 |
| SP2 | MAESTRO | 0.77 | 0.56 | 0.58 | 0.85 | 0.84 | 0.41 | 0.81 |
| | MAESTRO No S.A. | 0.73 | 0.52 | 0.49 | 0.8 | 0.82 | 0.32 | 0.78 |
| | NN Agents | 0.76 | 0.54 | 0.56 | 0.84 | 0.83 | 0.39 | 0.80 |
| | SVM Agents | 0.78 | 0.36 | 0.67 | 0.93 | 0.80 | 0.37 | 0.81 |
| | MLR Agents | 0.62 | 0.83 | 0.40 | 0.54 | 0.90 | 0.33 | 0.75 |

Table S6: Agents binary classification results for the SP1 and SP2 data sets. [a]Results for mutations that stabilize the structures. [b]Results for mutations with a destabilizing effect. [c]Blind test on protein level with respect on sequence similarities found by BLASTclust, as described above. [d]Blind test on protein level where we used the n-fold definition as provided by Pires *et al.* [5].

## ANN Ensemble Confidence Estimation

We performed n-fold cross validation experiments using an ensemble of seven ANNs instead of the seven MAESTRO agents for deriving the confidence estimation. Three of these ANNs are used as general agents, trained on the whole training set and the remaining four ANNs are trained on either stabilizing or destabilizing mutations. To overcome side effects by the fold definition, the folds are defined in the same way as for the results shown in Figure 3 of the main text. As shown in Figure S2, a lower prediction error can still be expected with higher estimated confidence, but the estimation is less reliable as the estimation based on the three different methods (ANN, SVM and MLR). The predictions error increases for high confidence values and more correct predictions receive a low confidence, compared to MAESTRO results.

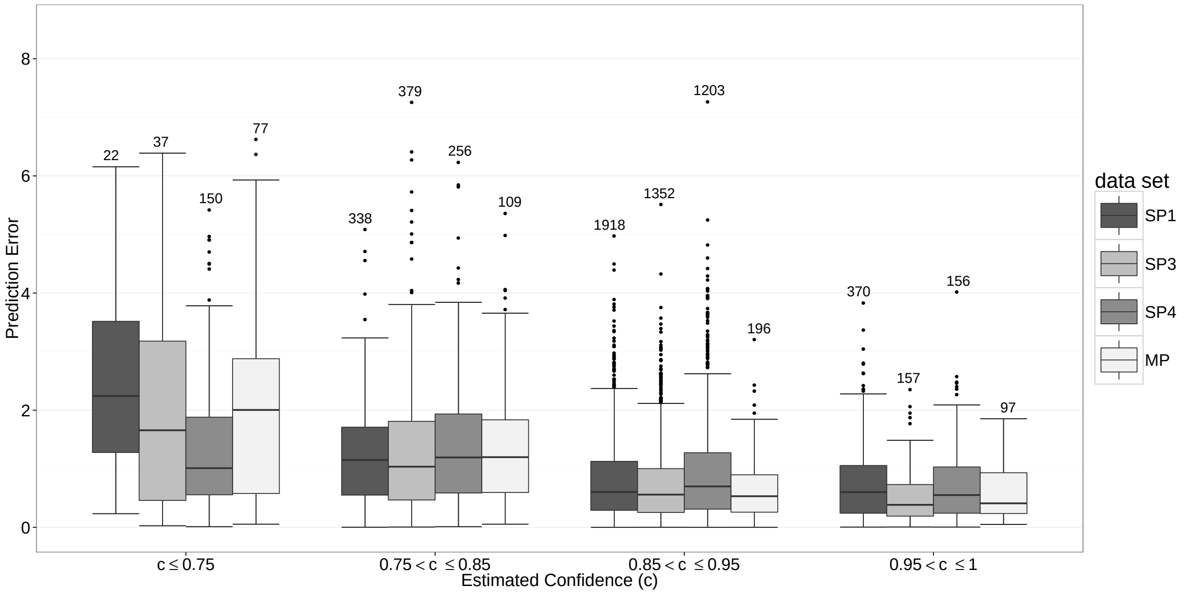


Figure S2: Confidence estimation and prediction error in case of an ANN ensemble. In contrast to the results presented in Figure 3 of the main text, in this experiment seven ANNs were utilized for the prediction. The figure shows the deviation between experimental determined ΔΔG values and the predictions for different confidence value ranges. The prediction error is defined as the absolute difference between the experimental determined ΔΔG and the predicted ΔΔG. Data are given for the three main single point mutation sets (SP1, SP3, SP4) as well as the multi-point mutation set (MP). The numbers of prediction per group are shown at the top.

# 5 Disulfide Bond Prediction

MAESTRO provides a special scan mode for disulfide bridges. Below we show the prediction performance on the SS1 set provided by Salam *et al.* [6]. The set includes 75 single chain X-ray structures with a resolution of 1.5$\AA$ or better. Each structure contains exactly one disulfide bridge.

For the prediction experiments the cysteine residues responsible for the disulfide bonds were exchanged to alanine by simply keeping the main chain and $C^{\beta}$ coordinates, removing the $S^{\gamma}$ and changing the residue type to ALA in the PDB file.

Table S7 shows the prediction results of the MAESTRO ΔΔG prediction as well as the results based on the MAESTRO-Score in comparison with the results reported by Salam *et al.*. In contrast to the method of Salam *et al.* MAESTRO was not particularly trained on disulfide bridge data. Still in 13 cases MAESTRO ranked the native bond on top compared to 15 cases of Salam's method.

| | | **MAESTRO** | | | | **MAESTRO-Score** | | | | **Salam** |
|---|---|---|---|---|---|---|---|---|---|---|
| **PDB** | **SS-** | **PDB struct.**[a] | | **minimized**[b] | | **PDB struct.**[a] | | **minimized**[b] | | ***et al.*** |
| **ID** | **Bridge** | $r_{abs}$[c] | $r_{rel}$[d] | $r_{abs}$[c] | $r_{rel}$[d] | $r_{abs}$[c] | $r_{rel}$[d] | $r_{abs}$[c] | $r_{rel}$[d] | **FRO**[e] |
| 1ABA | 14 / 17 | 1 | 0.03 | **0** | **0.00** | 4 | 0.1 | 1 | 0.02 | **0.00** |
| 1C7K | 99 / 112 | 1 | 0.02 | 3 | 0.04 | 8 | 0.12 | 12 | 0.17 | **0.00** |
| 1DYQ | 96 / 106 | 16 | 0.12 | 5 | 0.04 | 23 | 0.18 | 12 | 0.09 | 0.05 |
| 1GV9 | 198 / 238 | 14 | 0.09 | 103 | 0.63 | 33 | 0.21 | 125 | 0.76 | 0.04 |
| 1KNG | 92 / 95 | 6 | 0.09 | 23 | 0.29 | 12 | 0.18 | 39 | 0.49 | 0.02 |
| 1LF7 | 76 / 168 | 4 | 0.04 | 1 | 0.01 | 8 | 0.08 | 3 | 0.03 | 0.07 |
| 1LJU | 82 / 89 | 1 | 0.02 | 3 | 0.04 | 1 | 0.02 | 2 | 0.03 | 0.17 |
| 1M40 | 77 / 123 | 13 | 0.08 | 50 | 0.34 | 27 | 0.17 | 91 | 0.63 | 0.17 |
| 1MF7 | 128 / 318 | 7 | 0.06 | – | – | 20 | 0.18 | – | – | 0.09 |
| 1MJN | 161 / 299 | 5 | 0.05 | 37 | 0.36 | 11 | 0.11 | 45 | 0.44 | 0.03 |
| 1NKO | 46 / 106 | 6 | 0.09 | 9 | 0.13 | 6 | 0.09 | 15 | 0.22 | 0.02 |
| 1OAL | 52 / 147 | 3 | 0.03 | 2 | 0.02 | **0** | **0.00** | 4 | 0.04 | 0.03 |
| 1OLR | 6 / 35 | 23 | 0.15 | 27 | 0.17 | 41 | 0.26 | 44 | 0.28 | **0.00** |
| 1P3C | 32 / 48 | 12 | 0.08 | 22 | 0.14 | 26 | 0.17 | 42 | 0.26 | 0.06 |
| 1QGV | 38 / 79 | 6 | 0.1 | 2 | 0.03 | 5 | 0.08 | 3 | 0.05 | 0.35 |
| 1QK8 | 40 / 43 | **0** | **0.00** | **0** | **0.00** | **0** | **0.00** | 4 | 0.06 | 0.02 |
| 1R26 | 30 / 33 | 3 | 0.06 | 4 | 0.08 | 3 | 0.06 | 6 | 0.12 | 0.05 |
| 1RIE | 144 / 160 | 11 | 0.15 | 12 | 0.16 | 22 | 0.3 | 24 | 0.32 | 0.62 |
| 1SHU | 39 / 218 | 18 | 0.19 | 9 | 0.08 | 23 | 0.24 | 15 | 0.13 | 0.01 |
| 1T2I | 7 / 96 | 7 | 0.17 | 5 | 0.11 | 13 | 0.31 | 10 | 0.23 | **0.00** |
| 1T2J | 22 / 92 | 11 | 0.13 | 24 | 0.31 | 18 | 0.22 | 23 | 0.3 | 0.09 |
| 1UNR | 60 / 77 | 14 | 0.3 | – | – | 7 | 0.15 | – | – | 0.06 |
| 1VHU | 111 / 154 | 17 | 0.14 | 21 | 0.17 | 14 | 0.12 | 21 | 0.17 | 0.02 |
| 1WCU | 63 / 141 | 5 | 0.05 | 9 | 0.08 | 15 | 0.15 | 19 | 0.18 | 0.1 |
| 1XBU | 245 / 250 | 9 | 0.05 | 8 | 0.05 | 29 | 0.16 | 20 | 0.11 | 0.07 |
| 1XT5 | 26 / 109 | 7 | 0.08 | 13 | 0.15 | 11 | 0.13 | 13 | 0.15 | 0.05 |
| 1Y9L | 69 / 95 | 9 | 0.17 | 12 | 0.25 | 15 | 0.28 | 20 | 0.42 | **0.00** |
| 1ZK5 | 53 / 110 | 29 | 0.27 | 17 | 0.15 | 36 | 0.34 | 27 | 0.25 | **0.00** |
| 2A6Y | 151 / 185 | 14 | 0.1 | 4 | 0.03 | 22 | 0.16 | 17 | 0.12 | 0.1 |
| 2A6Z | 151 / 185 | 10 | 0.08 | 9 | 0.06 | 23 | 0.18 | 24 | 0.17 | 0.05 |
| 2AQM | 55 / 150 | 15 | 0.15 | 4 | 0.04 | 15 | 0.15 | 8 | 0.07 | 0.01 |
| 2CE0 | 67 / 73 | **0** | **0.00** | **0** | **0.00** | 1 | 0.02 | **0** | **0.00** | 0.18 |
| 2E0Q | 64 / 67 | 3 | 0.07 | 2 | 0.04 | 6 | 0.13 | 6 | 0.12 | 0.02 |
| 2ERF | 153 / 214 | 2 | 0.01 | 9 | 0.06 | 2 | 0.01 | 10 | 0.07 | 0.09 |
| 2FWG | 461 / 464 | **0** | **0.00** | 8 | 0.16 | 1 | 0.02 | 12 | 0.24 | 0.05 |
| 2HSH | 32 / 35 | 5 | 0.11 | 2 | 0.04 | 6 | 0.13 | 4 | 0.07 | 0.03 |

Continued on next page

| PDB ID | SS-Bridge | MAESTRO | | | | MAESTRO-Score | | | | Salam *et al.* |
|---|---|---|---|---|---|---|---|---|---|---|
| | | PDB struct.[a] | | minimized[b] | | PDB struct.[a] | | minimized[b] | | |
| | | $r_{abs}$[c] | $r_{rel}$[d] | $r_{abs}$[c] | $r_{rel}$[d] | $r_{abs}$[c] | $r_{rel}$[d] | $r_{abs}$[c] | $r_{rel}$[d] | FRO[e] |
| 2I1U | 37 / 40 | 3 | 0.06 | 5 | 0.09 | 2 | 0.04 | 12 | 0.21 | **0.00** |
| 2I4A | 32 / 35 | **0** | **0.00** | 4 | 0.07 | 6 | 0.13 | 5 | 0.09 | 0.03 |
| 2ICC | 22 / 94 | 5 | 0.1 | 25 | 0.45 | 4 | 0.08 | 26 | 0.46 | **0.00** |
| 2NWF | 134 / 151 | 7 | 0.07 | 18 | 0.2 | 25 | 0.26 | 37 | 0.42 | 0.15 |
| 2P39 | 95 / 113 | 34 | 0.44 | 21 | 0.26 | 34 | 0.44 | 24 | 0.29 | 0.07 |
| 2P52 | 173 / 239 | 4 | 0.04 | 6 | 0.06 | 12 | 0.12 | 21 | 0.2 | 0.03 |
| 2PY0 | 129 / 142 | 9 | 0.15 | 46 | 0.79 | 9 | 0.15 | 45 | 0.78 | 0.07 |
| 2QO4 | 80 / 91 | **0** | **0.00** | **0** | **0.00** | 1 | 0.02 | **0** | **0.00** | 0.07 |
| 2RKQ | 48 / 54 | 11 | 0.1 | 11 | 0.11 | 23 | 0.21 | 25 | 0.24 | 0.01 |
| 2VYO | 22 / 215 | 10 | 0.1 | 14 | 0.15 | 24 | 0.24 | 24 | 0.26 | 0.01 |
| 2XFD | 90 / 101 | 1 | 0.01 | 4 | 0.06 | 7 | 0.1 | 9 | 0.13 | **0.00** |
| 2YXF | 25 / 80 | **0** | **0.00** | **0** | **0.00** | **0** | **0.00** | **0** | **0.00** | 0.06 |
| 3CB9 | 147 / 204 | 4 | 0.03 | 13 | 0.1 | 11 | 0.08 | 44 | 0.33 | 0.04 |
| 3E8T | 8 / 15 | 1 | 0.01 | 4 | 0.03 | 4 | 0.04 | 20 | 0.17 | 0.03 |
| 3EDI | 42 / 198 | 4 | 0.04 | 1 | 0.01 | 8 | 0.08 | 5 | 0.04 | 0.01 |
| 3FSA | 3 / 26 | **0** | **0.00** | 2 | 0.03 | 5 | 0.07 | 12 | 0.15 | **0.00** |
| 3FZ4 | 10 / 13 | 3 | 0.06 | 2 | 0.04 | 4 | 0.08 | 4 | 0.07 | 0.05 |
| 3GA4 | 55 / 58 | 3 | 0.05 | 10 | 0.16 | 4 | 0.07 | 21 | 0.33 | 0.02 |
| 3GNZ | 37 / 63 | 7 | 0.06 | 1 | 0.01 | 22 | 0.19 | 18 | 0.14 | **0.00** |
| 3GUI | 21 / 142 | 3 | 0.05 | 8 | 0.11 | 1 | 0.02 | 8 | 0.11 | 0.04 |
| 3HNB | 2174 / 2326 | 5 | 0.05 | 14 | 0.13 | 14 | 0.15 | 30 | 0.28 | 0.03 |
| 3HZ8 | 57 / 60 | 8 | 0.09 | 5 | 0.06 | 12 | 0.13 | 10 | 0.11 | 0.04 |
| 3KFF | 64 / 157 | **0** | **0.00** | 2 | 0.03 | **0** | **0.00** | 4 | 0.05 | 0.05 |
| 3L4R | 64 / 157 | 5 | 0.06 | 6 | 0.06 | 7 | 0.08 | 9 | 0.1 | **0.00** |
| 3M1W | 5 / 64 | **0** | **0.00** | 4 | 0.03 | 10 | 0.07 | 13 | 0.08 | 0.07 |
| 3O22 | 89 / 186 | **0** | **0.00** | 10 | 0.1 | 2 | 0.02 | 13 | 0.14 | 0.03 |
| 3RT2 | 27 / 153 | 10 | 0.11 | 39 | 0.43 | 25 | 0.28 | 62 | 0.69 | 0.06 |
| 3RXW | 68 / 237 | 46 | 0.29 | 7 | 0.04 | 67 | 0.42 | 23 | 0.14 | 0.02 |
| 3SEB | 93 / 113 | **0** | **0.00** | 4 | 0.03 | 1 | 0.01 | 20 | 0.16 | 0.19 |
| 3SH4 | 159 / 193 | 3 | 0.02 | 13 | 0.09 | 6 | 0.04 | 20 | 0.14 | 0.04 |
| 3T0V | 23 / 88 | 15 | 0.21 | 25 | 0.32 | 16 | 0.22 | 27 | 0.34 | 0.16 |
| 3TPK | 22 / 96 | 8 | 0.11 | 13 | 0.16 | 9 | 0.13 | 13 | 0.16 | **0.00** |
| 3VOR | 106 / 170 | 6 | 0.05 | 21 | 0.17 | 20 | 0.18 | 26 | 0.21 | 0.05 |
| 3ZYP | 22 / 52 | 8 | 0.06 | 69 | 0.46 | 35 | 0.25 | 116 | 0.78 | 0.02 |
| 4EQ8 | 7 / 148 | 4 | 0.04 | 5 | 0.05 | 12 | 0.12 | 13 | 0.13 | 0.03 |
| 4F0W | 7 / 148 | 3 | 0.03 | 8 | 0.08 | 14 | 0.14 | 12 | 0.12 | **0.00** |
| 4FH4 | 77 / 123 | 6 | 0.04 | 80 | 0.47 | 14 | 0.08 | 115 | 0.68 | 0.04 |
| 4FTF | 74 / 111 | **0** | **0.00** | **0** | **0.00** | **0** | **0.00** | 2 | 0.05 | **0.00** |
| 4HWM | 68 / 124 | **0** | **0.00** | 3 | 0.04 | 1 | 0.02 | 5 | 0.07 | **0.00** |
| **Average** | | **7.2** | **0.08** | **13.5** | **0.13** | **13** | **0.13** | **22.1** | **0.21** | **0.06** |
| **Median** | | **5** | **0.06** | **8** | **0.08** | **11** | **0.13** | **15** | **0.15** | **0.03** |

Table S7: Disulfide bridge prediction performance on the SS1 data set, in comparison to the results reported by Salam *et al.* [6]. Results are shown for MAESTRO ΔΔG prediction and SSFs (MAESTRO-Score). [a]Results on original PDB structures. [b]Results on minimized structures. In the cases of 1MF7 and 1UNR, the $C^{\beta}$ distance become slightly larger than the cutoff distance of $5\AA$, after the minimization. [c]The absolute rank $r_{abs}$ is given in the range of 0 (top) to $n-1$. [d]The relative rank $r_{rel}$ is defined as $r_{abs}/(n-1)$. [e]Data obtained from Salam *et al.*.

## 6 Mutation Scan

MAESTRO provides three scan methods: optimal, greedy, and EA (Evolutionary Algorithm) for the search of combinations of point mutations which stabilize or destabilize a structure as much as possible. Below, we compare the performance of the three approaches. All experiments were performed on eight randomly selected PDB structures as well as two structures with a sequence length of exactly 30 residues.

As the optimal search is potentially very time consuming, we set up a first experiment, in which the number of allowed mutation sites was limited to 30 and the number of mutations points was set to three. The mutation sites were randomly selected. Scans for the most stabilizing and the most destabilizing mutants were performed.

As shown in Table S8, the scan methods behave very similar in the case of a small number of allowed mutation sites and a small number of maximum substitutions. Only in two cases, the greedy search performs marginally worse than the other methods.

| | | **Method** | | |
|---|---|---|---|---|
| **PDB** | **Scan**[a] | **Optimal**[b] | **Greedy**[b] | **EA**[b] |
| 1fxf | stabilize | 1.000 | 1.000 | 1.000 |
| | destabilize | 1.000 | 1.000 | 1.000 |
| 1q2u | stabilize | 1.000 | 0.998 | 1.000 |
| | destabilize | 1.000 | 0.999 | 1.000 |
| 1urw | stabilize | 1.000 | 1.000 | 1.000 |
| | destabilize | 1.000 | 1.000 | 1.000 |
| 2ds1 | stabilize | 1.000 | 1.000 | 1.000 |
| | destabilize | 1.000 | 1.000 | 1.000 |
| 2ph8 | stabilize | 1.000 | 1.000 | 1.000 |
| | destabilize | 1.000 | 1.000 | 1.000 |
| 3ati | stabilize | 1.000 | 1.000 | 1.000 |
| | destabilize | 1.000 | 1.000 | 1.000 |
| 3loe | stabilize | 1.000 | 1.000 | 1.000 |
| | destabilize | 1.000 | 1.000 | 1.000 |
| 4bfh | stabilize | 1.000 | 1.000 | 1.000 |
| | destabilize | 1.000 | 1.000 | 1.000 |
| 4gpr | stabilize | 1.000 | 1.000 | 1.000 |
| | destabilize | 1.000 | 1.000 | 1.000 |
| 4kfj | stabilize | 1.000 | 1.000 | 1.000 |
| | destabilize | 1.000 | 1.000 | 1.000 |

Table S8: Performance comparison of the three mutation scan methods. Limited to 30 mutation sites and three mutation points. [a]Scan mode, either the search for the most destabilizing or the most stabilizing set of point mutations. [b]The performance is given relative to the optimal search.

In a second experiment the number of mutation sites was not limited and the number of maximum substitutions was set to either 3, 5, or 10. For runtime reasons this experiment was only performed with the greedy and EA search.

As shown in Table S9, the results are more divergent than in the experiment before. In most cases, the EA performs better than the greedy search.

| PDB | Mutation sites[a] | Scan[b] | 3 substitutions Greedy[c] | 3 substitutions EA[c] | 5 substitutions Greedy[c] | 5 substitutions EA[c] | 10 substitutions Greedy[c] | 10 substitutions EA[c] |
|---|---|---|---|---|---|---|---|---|
| 1fxf | 124 | stabilize | 1.000 | 1.000 | 0.872 | 1.000 | 0.812 | 1.000 |
| | | destabilize | 0.962 | 1.000 | 0.827 | 1.000 | 0.956 | 1.000 |
| 1q2u | 189 | stabilize | 1.000 | 0.944 | 0.979 | 1.000 | 1.000 | 0.745 |
| | | destabilize | 1.000 | 0.996 | 1.000 | 0.999 | 0.970 | 1.000 |
| 1urw | 274 | stabilize | 0.777 | 1.000 | 0.826 | 1.000 | 0.942 | 1.000 |
| | | destabilize | 0.986 | 1.000 | 1.000 | 0.959 | 1.000 | 0.948 |
| 2ds1 | 290 | stabilize | 1.000 | 0.982 | 1.000 | 0.966 | 1.000 | 0.942 |
| | | destabilize | 0.951 | 1.000 | 0.959 | 1.000 | 1.000 | 0.921 |
| 2ph8 | 365 | stabilize | 0.999 | 1.000 | 1.000 | 0.942 | 0.774 | 1.000 |
| | | destabilize | 0.872 | 1.000 | 0.890 | 1.000 | 0.961 | 1.000 |
| 3ati | 223 | stabilize | 1.000 | 0.994 | 1.000 | 0.960 | 1.000 | 0.914 |
| | | destabilize | 0.971 | 1.000 | 0.973 | 1.000 | 0.952 | 1.000 |
| 3loe | 30 | stabilize | 0.749 | 1.000 | 0.717 | 1.000 | 0.800 | 1.000 |
| | | destabilize | 1.000 | 1.000 | 1.000 | 0.999 | 1.000 | 0.997 |
| 4bfh | 30 | stabilize | 0.833 | 1.000 | 0.864 | 1.000 | 0.939 | 1.000 |
| | | destabilize | 0.963 | 1.000 | 0.944 | 1.000 | 0.992 | 1.000 |
| 4gpr | 149 | stabilize | 0.938 | 1.000 | 0.949 | 1.000 | 0.952 | 1.000 |
| | | destabilize | 1.000 | 1.000 | 1.000 | 0.991 | 1.000 | 0.916 |
| 4kfj | 259 | stabilize | 0.923 | 1.000 | 0.923 | 1.000 | 0.935 | 1.000 |
| | | destabilize | 0.889 | 1.000 | 0.846 | 1.000 | 0.735 | 1.000 |
| | | Average: | 0.941 | 0.996 | 0.928 | 0.991 | 0.936 | 0.969 |
| | | Std.dev.: | 0.078 | 0.013 | 0.080 | 0.018 | 0.084 | 0.062 |

Table S9: Performance comparison of the three mutation scan methods. Limited to three, five or ten substitutions. [a]Number of mutation sites in the structures. [b]Scan mode, either the search for the most destabilizing or the most stabilizing set of point mutations. [c]The performance is given relative to the best result (per n substitutions).

The runtime of a scan depends strongly on the chosen method, the number of mutations sites, the structure size and the number of maximum substitutions. In the first experiment, the optimal search had a runtime between six hours and five days, while the EA run took between 30 and 85 minutes and the greedy search was finished after 40 seconds and two minutes.

In the second experiments we observed that the greedy algorithm strongly depends on the number of maximum substitutions, as expected. While in the first experiment the maximum runtime was about two minutes, the maximum runtime increased to 35 minutes in case of a maximum of five substitutions and to 80 minutes in case of a maximum of ten substitutions. In contrast to that, the runtime of the EA algorithm was only slightly affected and not longer than 90 minutes.

For all these reasons, we recommend the optimal search only for small structures or a small set of allowed substitutions. The greedy search is, in most cases, faster than the EA variant, but the EA provides better results in many cases and a more stable runtime. Thus, from our point of view, the EA will be the best choice for most use cases.

# 7 Misclassified ProTherm Entries

On the ProTherm web page in the "Known Problems" section, the database maintainers hint to the "Sign convention for free energy change" and they claim that they are not able to check whether submitting authors did fully comply with the given conventions. Therefore, users of ProTherm should cross-check the ΔΔG values. As mentioned in the main results, we found some serious classification errors in the data set provided by Tian *et al.* [8]. We then took three samples from the Tian set, the ten most destabilizing mutants, the ten most stabilizing, as well as a random sample of 100 mutants of the remaining data set. In these samples we found eight entries which are misclassified.

| **ProTherm entry** | **PDB ID** | **Mutation** | **Wrong ΔΔG/class** | | **Reference** |
|---|---|---|---|---|---|
| 12235 | 1OH0 | Y16S | 11.90 | (stabilizing) | Nam *et al.* [3] |
| 12236 | 1OH0 | Y32S | 13.70 | (stabilizing) | Nam *et al.* [3] |
| 12237 | 1OH0 | Y57S | 9.50 | (stabilizing) | Nam *et al.* [3] |
| 15807 | 1FKJ | W59F | −2.72 | (destabilizing) | Fulton *et al.* [2] |
| 17632 | 1TIT | L60A | 5.27 | (stabilizing) | Fowler *et al.* [1] |
| 17628 | 1TIT | V13A | 2.37 | (stabilizing) | Fowler *et al.* [1] |
| 16141 | 1RX4 | G95A | 1.30 | (stabilizing) | Svensson *et al.* [7] |
| 10581 | 1BTA | L34V | 1.10 | (stabilizing) | Nölting *et al.* [4] |

Table S10: Sign error examples.

The first consequence was, that we were not able to compare our prediction results with the work of Tian *et al.* [8]. The second consequence was the retrieval of our own sets SP4 and MP, where we cross check the ΔΔG in ProTherm values with literature. Although we still cannot claim that there are no sign and value errors in our sets at least some errors have been resolved.

# 8 Statistical Scoring Function Parameters

| Parameter | Value |
|---|---|
| $C^\alpha - C^\alpha$ pair SSF | |
| Lower distance cutoff | $0.0\AA$ |
| Upper distance cutoff | $19.0\AA$ |
| Bins | 95 |
| $\sigma$ | $0.6\AA$ |
| $C^\beta - C^\beta$ pair SSF | |
| Lower distance cutoff | $1.0\AA$ |
| Upper distance cutoff | $11.0\AA$ |
| Bins | 50 |
| $\sigma$ | $0.8\AA$ |
| $C^\alpha$ contact SSF | |
| Contact radius | $10.0\AA$ |
| Maximum counts | 100 |
| Bins | 7 |
| $\sigma$ | 1.4 contact counts |

The contribution of single measurements were smoothed with a Gaussian kernel. The corresponding values of $\sigma$ are given in the above table.

# References


[1] Susan B. Fowler, Robert B. Best, José L. Toca Herrera, Trevor J. Rutherford, Annette Steward, Emanuele Paci, Martin Karplus, and Jane Clarke. Mechanical unfolding of a titin Ig domain: structure of unfolding intermediate revealed by combining AFM, molecular dynamics simulations, NMR and protein engineering. *J Mol Biol*, 322(4):841–849, Sep 2002.

[2] Kate F. Fulton, Sophie E. Jackson, and Ashley M. Buckle. Energetic and structural analysis of the role of tryptophan 59 in FKBP12. *Biochemistry*, 42(8):2364–2372, Mar 2003.

[3] G. H. Nam, D. S. Jang, S. S. Cha, T. H. Lee, D. H. Kim, B. H. Hong, Y. S. Yun, B. H. Oh, and K. Y. Choi. Maintenance of alpha-helical structures by phenyl rings in the active-site tyrosine triad contributes to catalysis and stability of ketosteroid isomerase from Pseudomonas putida biotype B. *Biochemistry*, 40(45):13529–13537, Nov 2001.

[4] B. Nölting, R. Golbik, and A. R. Fersht. Submillisecond events in protein folding. *Proc Natl Acad Sci U S A*, 92(23):10668–10672, Nov 1995.

[5] Douglas E V. Pires, David B. Ascher, and Tom L. Blundell. mCSM: predicting the effects of mutations in proteins using graph-based signatures. *Bioinformatics*, 30(3):335–342, Feb 2014.

[6] Noeris K Salam, Matvey Adzhigirey, Woody Sherman, and David A Pearlman. Structure-based approach to the prediction of disulfide bonds in proteins. *Protein Eng Des Sel*, May 2014.

[7] Anna-Karin E. Svensson, John C O'Neill, Jr, and C Robert Matthews. The coordination of the isomerization of a conserved non-prolyl cis peptide bond with the rate-limiting steps in the folding of dihydrofolate reductase. *J Mol Biol*, 326(2):569–583, Feb 2003.

[8] Jian Tian, Ningfeng Wu, Xiaoyu Chu, and Yunliu Fan. Predicting changes in protein thermostability brought about by single- or multi-site mutations. *BMC Bioinformatics*, 11:370, 2010.
